# Modulation of surface response in a single plasmonic nanoresonator

**DOI:** 10.1126/sciadv.adn5227

**Published:** 2024-09-06

**Authors:** Luka Zurak, Christian Wolff, Jessica Meier, René Kullock, N. Asger Mortensen, Bert Hecht, Thorsten Feichtner

**Affiliations:** ^1^ Nano-Optics and Biophotonics Group, Experimental Physics 5, Institute of Physics, University of Würzburg, Germany.; ^2^ POLIMA–Center for Polariton-driven Light-Matter Interactions, University of Southern Denmark, Campusvej 55, DK-5230 Odense M, Denmark.; ^3^ Danish Institute for Advanced Study, University of Southern Denmark, Campusvej 55, DK-5230 Odense M, Denmark.

## Abstract

Scattering of light by plasmonic nanoparticles is classically described using bulk material properties with infinitesimally thin boundaries. However, because of the quantum nature of electrons, real interfaces have finite thickness, leading to nonclassical surface effects that influence light scattering in small particles. Electrical gating offers a promising route to control and study these effects, as static screening charges reside at the boundary. We investigate the modulation of the surface response upon direct electrical charging of single plasmonic nanoresonators. By analyzing measured changes in light scattering within the framework of surface response functions, we find the resonance shift well accounted for by modulation of the classical in-plane surface current. Unexpectedly, we also observed a change in the resonance width, indicating reduced losses for negatively charged resonators. This effect is attributed to a nonclassical out-of-plane surface response, extending beyond pure spill-out effects. Our experiments pave the way for electrically driven plasmonic modulators and metasurfaces, leveraging control over nonclassical surface effects.

## Teaser

Electrical control over the nonclassical surface response enables light modulation, pointing toward ultrafast active plasmonics.

## INTRODUCTION

Localized plasmonic resonances, coupled states of photons and electrons in nanostructured metals, have been extensively studied and widely used to control light-matter interaction (*1*–*9*). However, the active tuning of plasmonic resonances still remains a major obstacle for the realization of integrated high-speed optical modulators and tunable metamaterials (*10*). One of the most compelling pathways toward high-speed dynamic tuning is via the density of the metal’s electron gas. While this idea has already been exploited in dilute systems, such as graphene (*11*–*13*), it is more difficult to realize in metallic nanoresonators due to their orders of magnitude larger carrier densities. Various methods to increase the capacitance have therefore been used, such as embedding metallic nanoresonators in a chemical reductant or ion gel (*14*–*24*). However, these approaches have severe limitations, such as slow operation [up to few kilohertz in (*23*)] and inconclusive results. Some of them exhibit hysteresis, likely due to double-layer formation and/or electrochemical reactions at the metal surface (*25*), suggesting origins of the observed tuning effects beyond pure charging. Consequently, there is a need for experiments free from the potential impact of environmental variations by means of direct electrical charging.

On the theoretical side a common approach of modeling the impact of charging effects on light scattering is based on the incorrect assumption that electrostatic charges lead to a change in the bulk properties of metallic nanoresonators (*15*, *16*, *26*). Only few studies have explored the impact of surface charges on light scattering (*20*, *22*, *27*–*29*). On one side, classical models predict resonance blue shifts for negatively charged systems and only a minor broadening of the resonance width. In contrast, using a quantum approach, Zapata Herrera *et al.* investigated small spheres with time-dependent density functional theory (DFT) and predicted a resonance red shift accompanied by a resonance broadening in negatively charged systems (*28*). This is supported by a study of Li *et al.* (*29*) who used a quantum hydrodynamic model to theoretically examine the response of a nanoscale electron reservoir placed within a narrow plasmonic gap, promising a large scattering modulation. However, the computationally demanding quantum approaches can only treat either small systems [≈5 nm in (*28*)] or highly symmetric larger ones (*29*). These practical limitations of quantum approaches together with their contrasting predictions compared to classical studies highlight the demand for an easily accessible and universal theoretical approach. This would allow to identify the origins of charge-induced spectral changes for arbitrarily shaped plasmonic nanoresonators.

Recently, studying the optical response of electrons at metal surfaces has gained much attention, as nonclassical surface properties have been found to strongly shape the response of nanoscale plasmonic systems (*30*–*41*). Reduced field enhancement, accompanied by broadening and spectral shifting of resonances, are caused by electron spill-out (*39*, *42*), nonlocality (*43*), and Landau damping (*44*–*46*), effects that can be treated using the universal framework of surface response functions, also known as Feibelman *d*-parameters (*30*, *40*). These can be readily added to framework of classical electrodynamics by modifying the boundary conditions (*40*).

Therefore, conducting systematic and reliable experiments using direct electrical charging and evaluating the results within the framework of surface response functions will deepen the understanding of charge-induced surface effects. Thanks to its practicality, this approach can ultimately be leveraged to optimize electrical tuning of plasmonic resonances in smaller systems where surface effects play a dominant role (*28*, *29*). Furthermore, this is particularly important for the plasmoelectric potential, an effect that fundamentally depends on the strength of electrical tunability in plasmonic resonances (*26*).

**Fig. 1.**
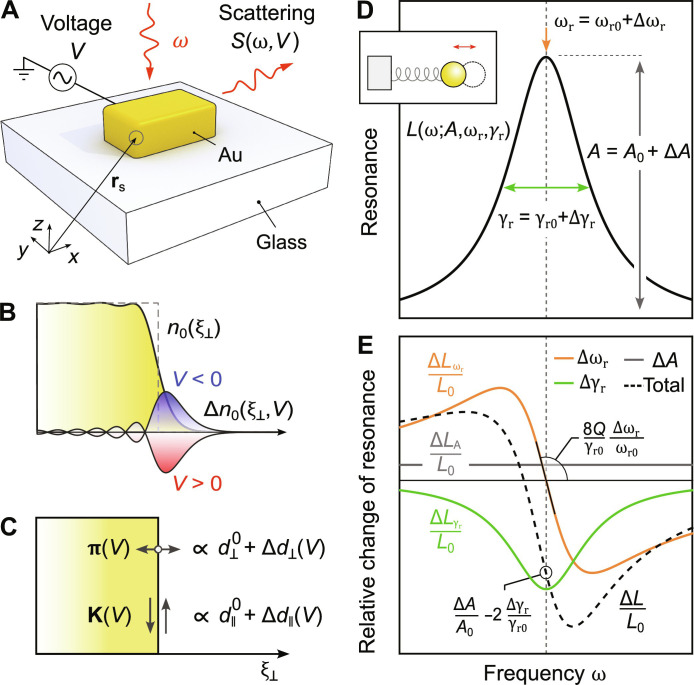
Influence of electrostatically induced surface charges on the optical response of plasmonic nanoresonators. (**A**) Sketch of the studied system; a rectangular gold (Au) nanoresonator is placed on top of a glass substrate. The applied electric potential *V* induces a change in the resonance, which is detected by recording the scattering signal *S*(ω, *V*) as a function of the photon energy ℏω and the applied potential. (**B**) Equilibrium and induced electron density distributions at the gold-vacuum interface calculated using a Wigner-Seitz radius of 3.18a_0_ in the jellium description (*a*_0_ is the Bohr radius). Gray dashed line denotes the position of a positive background. (**C**) The change in electron density at the surface position **r**_s_ perturbs the *d*-parameters and, in turn, the optical response of the system by altering the surface polarization **P**_s_(*V*) (see Eq. 2). (**D**) Model of a harmonic oscillator and a corresponding resonance curve with a Lorentzian line shape *L*(ω; *A*, ω_r_, γ_r_) described by its amplitude *A*, resonance frequency ω_r_, and width γ_r_. (**E**) Small perturbations of Δ*A* > 0, Δω_r_ < 0, or Δγ_r_ > 0 lead to distinct relative changes of the resonance represented by the gray line (Δ*A*), the orange curve (Δω_r_), and the green curve (Δγ_r_), respectively. Change in resonance width and amplitude is related to relative change at the resonance frequency, while change in resonance position is linked to the slope of this relative change; *Q* = ω_r_/γ_r_ stands for the quality factor. Total change, represented by the black dashed line, is a superposition of the three.

In this study, we experimentally investigate the effect of direct electrical charging on the fundamental dipolar resonance of single plasmonic nanoresonators (sketched in Fig. 1A). To this end, we measure the voltage-induced relative change in light scattering spectra for applied electric potentials of up to ±20 V, from which we determine the induced changes in the resonance amplitude and eigenfrequency. Our choice of geometry, experimentally realized with high accuracy, enables quantitative comparisons of numerical results with high-precision measurements. We analyze the observed effects within the general framework of surface response functions and develop a model taking into account electron spill-out within the local response approximation (LRA). We find that, unlike the surface response in charge-neutral plasmonic nanoresonators, dominated by a nonclassical out-of-plane response [see, e.g., (*40*)], our charged interfaces also exhibit a strong in-plane response showing a classical character. Contrasting expectations in the spectral changes of in-plane and out-of-plane response enable us to attribute the observations to either classical or nonclassical origins of the surface response.

While a large part of the measured shift in the resonance frequency can be accounted for by the in-plane surface response, this contribution alone cannot explain the observed change in the resonance width. This discrepancy increases when we include the out-of-plane response contributions based purely on spill-out effects in LRA. Therefore, the basic assumptions of our model need to be extended beyond the electron spill-out to include other nonclassical effects, such as anisotropy of the local permittivity and nonlocal effects hidden in the perpendicular component of the *d*-parameters. We show that already including phenomenological nonlocality substantially affects the out-of-plane response, reducing the mismatch between experiment and our modified LRA model. Regardless of the underlying model, we are able of providing an estimation for the size of the out-of-plane component.

Last, our work represents the first step toward ever smaller plasmonic modulators with direct electrical control. Ultimately, we anticipate this will result in ultrafast devices with large modulation depths, shaped by the nonclassical surface effects.

## RESULTS

### Surface response functions

Bohren and Hunt (*27*) made an initial attempt to model how adding electrons to the surface of a plasmonic nanoresonator affects its resonance. They assumed that, in the picture of classical electrodynamics, added electrons modify the surface conductivity σ_s_ and contribute to an in-plane surface current **K** = σ_s_**E**_∥_ which is driven by the tangential component of the electric field **E**_∥_. This results in a change of the effective plasma frequency (see section S1.1).

However, to fully capture the optical response of electrons at the surface one has to also take into account various nonclassical effects. First of all, electron spill-out causes the surface to acquire a finite thickness (see Fig. 1B), which leads to an out-of-plane response. Other quantum effects, which influence the electron response at the surface, are nonlocality, Landau damping and conduction through surface states (*47*, *48*). All such effects can be accounted for by Feibelman *d*-parameters (*30*, *47*). The *d*-parameters, *d*_⊥_ and *d*_∥_, are centroids of the induced charge density and of the normal derivative of the tangential current, respectively (see section S1.2) (*30*). Hence, as demonstrated by Christensen *et al.* (*49*), *d*-parameters describe the surface polarization

$$P_{s}=\pi +i\omega^{-1}K$$ (1)

which can be added to the classical framework by modifying the boundary conditions (see section S1.3) (*40*). The out-of-plane electron oscillation, proportional to *d*_⊥_, is described via the dipole density $\pi =\varepsilon_{0}d_{⊥}\Delta E_{⊥}{\hat{\xi }}_{⊥}$ , where ε_0_ is the free-space permittivity, *E*_⊥_ is the perpendicular component of the electric field, and ${\hat{\xi }}_{⊥}$ is the unit vector normal to the surface (see Fig. 1C). The parallel component *d*_∥_ contributes to an in-plane surface current **K **= *i*ω*d*_∥_Δ**D**_∥_ as already discussed above, where **D**_∥_ is the tangential component of the displacement field.

If we assume a simple harmonic oscillator as a model for a resonance of plasmonic nanoresonator (see Fig. 1D), the associated Lorentzian *L*(ω; *A*, ω_r_, γ_r_) can be described by an amplitude *A* and a complex eigenfrequency ${\tilde{\omega }}_{r}=\omega_{r}-i\gamma_{r}/2$ (*50*). The real part corresponds to the resonance frequency, while the imaginary part describes damping of the system via radiation and ohmic losses. Surface effects, described by the Feibelman *d*-parameters, lead to a change of the plasmonic nanoresonator’s complex eigenfrequency $\Delta {\tilde{\omega }}_{r}$ , which is directly related to the surface polarization via the integral (*40*, *51*)

$$\Delta {\tilde{\omega }}_{r}=-{\tilde{\omega }}_{r0}\int_{s}‍E^{\left(0\right)}\cdot P_{\;s}^{\left(0\right)}\;ds$$ (2)

where s denotes the surface of the resonator. Here, $P_{s}^{\left(0\right)}$ represents the surface polarization **P**_s_ evaluated at the unperturbed eigenfrequency ${\tilde{\omega }}_{r0}$, with the unperturbed eigenfields **E**^(0)^ and **D**^(0)^ obtained under the assumption of classical, piecewise constant material properties, while neglecting any nonclassical effects. Therefore, the experimentally observed eigenfrequency of an uncharged plasmonic nanoresonator corrected by surface effects is ${\tilde{\omega }}_{r}={\tilde{\omega }}_{r0}+\Delta {\tilde{\omega }}_{r}$.

In the case of a controlled perturbation, an additional shift in the spectral position of the resonance frequency Δω_r_ causes a distinct pattern of the relative change of the resonance, as depicted in Fig. 1E. It can be easily differentiated from the patterns of relative change caused by changes of the width of the resonance Δγ_r_ and the amplitude Δ*A* (see section S1.4).

We use the formalism of *d*-parameters to investigate and describe the voltage-induced resonance changes of a single gold nanoresonator residing on top of a glass substrate (see Fig. 1A). At any point on the surface of the nanoresonator **r**_s_, an applied voltage *V* will introduce a change in the equilibrium electron density (see Fig. 1B) and locally perturb the surface response functions

$$d_{⊥,∥}\left(r_{s},V\right)=d_{⊥,∥}^{0}\left(r_{s}\right)+\Delta d_{⊥,∥}\left(r_{s},V\right)$$ (3)

where we suppress the ω-dependence for brevity. Here, $d_{⊥,∥}^{0}\left(r_{s}\right)$ are the unperturbed *d*-parameters, typically taken to be constant across a metal-dielectric interface. The *d*-parameter perturbations Δ*d*_⊥,∥_(**r**_s_, *V*) will lead to a local change of the surface polarization Δ**P**_s_(**r**_s_, *V*) (see Fig. 1C), which will introduce a voltage-induced change in the resonance amplitude Δ*A*(*V*), as well as the system’s complex eigenfrequency $\Delta {\tilde{\omega }}_{r}\left(V\right)$, in accordance with Eq. 2. These changes can be detected by recording the relative change of scattering spectrum, Δ*S*/*S*_0_(ω, *V*) (as shown in Fig. 1E), where Δ*S*(ω, *V*) is the voltage-induced change in scattering, and *S*_0_ = *S*(ω,0) is the scattering spectrum of the uncharged system. The scattering *S* is directly proportional to the scattering cross section σ_sca_.

### Experiment

**Fig. 2.**
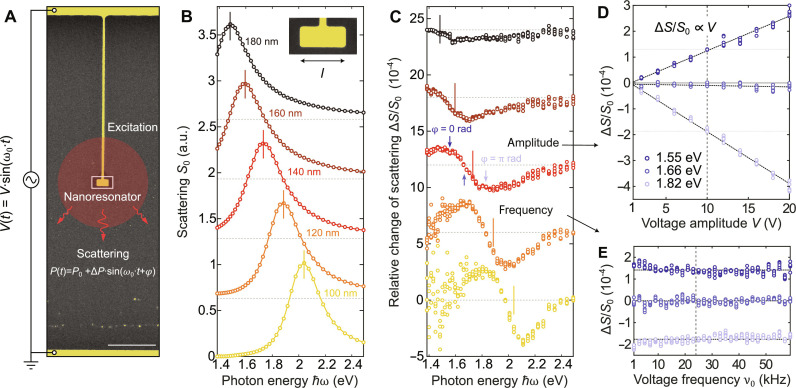
Measuring the voltage induced change of a plasmonic resonance. (**A**) Colored scanning electron microscopy (SEM) image of a single electrically connected gold nanoresonator. The structure is driven with a sinusoidal voltage *V*(*t*) while monitoring amplitude Δ*P* and phase ϕ of the scattered power. Scale bar, 500 nm. (**B**) Measured scattering spectra for resonators with length ranging from 180 down to 100 nm. The inset shows a colored close-up SEM image of the 180-nm-long nanoresonator. (**C**) Relative change of scattering as obtained from lock-in measurements for 10 V of voltage amplitude corresponding to the resonators in (A). (**D** and **E**) Dependency of relative change of scattering on the amplitude/frequency of the applied voltage, respectively, for three spectral points denoted in (C).

To experimentally investigate the effect of charging on the plasmonic resonance, we conducted dark-field scattering measurements on electrically connected nanoresonators, as depicted in Fig. 2A. The plasmonic resonators under investigation are fabricated from 50-nm-thick single-crystalline gold microplatelets (*52*), using a two-step focused ion beam (FIB) milling process [similar to the procedure in (*53*)]. The first step involved Ga-FIB to create the rough shape of the resonator and connector. Then, He-FIB was used to refine the structure’s final shape with high precision. An example of a 180-nm-long nanoresonator is shown in the inset of Fig. 2B. The resonance was recorded by spectrally tuning a supercontinuum white-light laser using a narrowband filter with polarization along the long-axis of the resonator (for more details, see sections S2.1 to S2.3). The scattering spectrum *S* is proportional to the ratio of the power scattered by the structure *P* and the incoming intensity *I* (see sections S2.3 and S2.4). In Fig. 2B, we plot the normalized scattering spectra of 80-nm-wide resonators with lengths ranging from 180 to 100 nm. To retrieve the change in scattering we drive the system using a sinusoidal voltage with an amplitude *V* at frequency ν_0_ and use a phase-sensitive lock-in amplifier to detect both the amplitude Δ*P* and the phase ϕ of the voltage-modulated scattered power, as illustrated in Fig. 2A. The unperturbed scattered power *P*_0_ was recorded using an optical powermeter placed in one of the branches of the detection system (see fig. S10). By repeating the measurement across the spectrum for a voltage amplitude of 10 V at a linear frequency of 24 kHz, we observe a purely in-phase lock-in signal (ϕ = 0, π) at the fundamental frequency for the relative change of scattering shown in Fig. 2C (see section S2.5).

For all nanoresonators, we consistently observe a qualitatively similar change in scattering, suggesting both a red shift and broadening of the resonance under a positive bias, i.e., exhibiting a blue shift and narrowing under a negative bias (see section S2.1). Notably, smaller structures demonstrate a more pronounced modulation indicating a route to further increase the effect. For the exemplary case of the 140-nm-long resonator, we demonstrate a linear relation between the observed changes and the applied voltage’s amplitude for signals at three specific spectral points (see Fig. 2D). In addition, we demonstrate that the observed changes at these three spectral points remain unaffected by a change in the AC signal frequency within the range constrained by the responsivity of the lock-in amplifier (see Fig. 2E). Furthermore, we observe similar spectral changes also with a thin coverage of AlO_x_ (see section S2.5). To get an insight into the mechanism which causes the experimentally observed resonance changes within the framework of surface response functions, we need to determine the voltage-dependent *d*-parameter perturbations (see Eq. 3).

### Electron spill-out and LRA

**Fig. 3.**
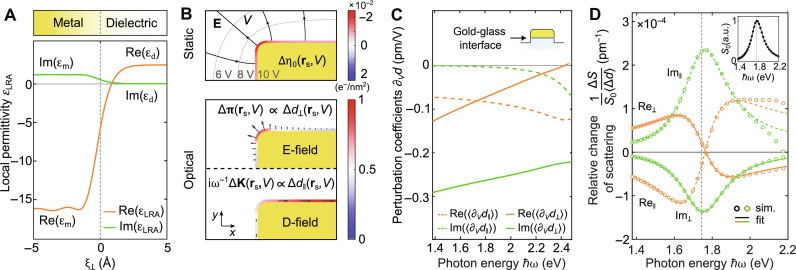
Influence of the *d*-parameter perturbations on a plasmonic resonance. (**A**) Spatial dependency of local permittivity at the gold-glass interface. (**B**) Top: Simulated static electric field lines at half of the antenna height (black arrowed lines) and contours of electric potential (gray dashed lines) for a 10-V equipotential at the surface of the structure. Excess electrons are nonuniformly distributed across the surface, represented by the induced surface electron density Δη_0_(**r**_s_,*V*) = *C*_s_(**r**_s_)*V*/*q*_e_ (surface color). Bottom: Simulated perturbed surface polarization Δ**P**_s_(**r**_s_, *V*) corresponding to an out-of-plane response Δ**π**(**r**_s_, *V*) (up) with electric field, and an in-plane response Δ**K**(**r**_s_, *V*) (down) with displacement field. (**C**) Spectral dependency of surface-averaged *d*-parameter perturbation coefficients for the gold-glass interface calculated with Eqs. 10 and 11. The *d*_⊥_ component contributions are depicted with solid lines, while those of the *d*_∥_ component are represented with dashed lines. Real parts are given in orange, and imaginary parts in green. (**D**) Calculated relative change of scattering Δ*S*/*S*_0_ per 1 pm of introduced interface-averaged *d*-parameter perturbations Δ*d*(**r**_s_). Each spectra is fitted using relative change of Lorentzian. The inset shows the calculated unperturbed scattering spectrum *S*_0_ fitted using Lorentzian modified with linear background.

A complete analysis of determining Δ*d*_⊥,∥_(**r**_s_, *V*) would encompass all possible microscopic phenomena taking place at the surface of the charged nanoresonator. However, as demonstrated in (*40*), efforts to predict even unperturbed *d*-parameters at gold interfaces so far have been in vain. Therefore, we follow the approach outlined in (*42*), where it has been demonstrated that large contributions to unperturbed *d*-parameters can be assigned to electron spill-out effects. Thus, in a first approximation, we calculate the perturbations Δ*d*_⊥,∥_(**r**_s_, *V*) resulting solely from the electron spill-out within the LRA, by considering only the first-order terms in the Taylor expansion

$$\Delta d_{⊥,∥}\left(r_{s},V\right)≃\frac{\partial_{V}d_{⊥,∥}\left(r_{s},V\right)}{\partial V}V$$ (4)

where we omit dependencies in ω and **r**_s_. We assume a position-dependent plasma frequency $\omega_{p}\left(\xi_{⊥},V\right)=\omega_{p0}\sqrt{n_{0}\left(\xi_{⊥},V\right)/n_{0}}$ , where ω_p0_ is the unperturbed plasma frequency, *n*_0_(ξ_⊥_, *V*) is the voltage-dependent equilibrium electron density, *n*_0_ is the bulk free electron density and ξ_⊥_ parameterizes the distance perpendicular to the interface to capture, e.g., the electron spill-out. In this approximation, the optical properties are described by a position-dependent local permittivity (see Fig. 3A)

$$\varepsilon_{\text{LRA}}\left(\xi_{⊥},V\right)=\varepsilon_{b}\left(\xi_{⊥}\right)-\frac{\omega_{p0}^{2}}{\omega^{2}+i\gamma \omega }\frac{n_{0}\left(\xi_{⊥}\right)+\Delta n_{0}\left(\xi_{⊥},V\right)}{n_{0}}$$ (5)

where *n*_0_(ξ_⊥_) is the equilibrium electron density of an uncharged system and Δ*n*_0_(ξ_⊥_, *V*) is the voltage-induced electron density (see Fig. 1B). ε_b_(ξ_⊥_) is the position-dependent background permittivity, which in bulk gold is dominated by interband contributions, while outside gold, it transitions to the permittivity of the surrounding dielectric. For simplicity, we assume that the electron response at any point at the surface is isotropic, although it is expected that the equation of motion perpendicular to the interface contains additional terms that describe the influence of the boundary. Within these assumptions, according to (*42*) the *d*-parameter perturbation coefficients can be expressed in integral form as

$$\frac{\partial d_{⊥}}{\partial V}=\frac{\varepsilon_{d}\varepsilon_{m}}{\varepsilon_{m}-\varepsilon_{d}}\int_{-\infty }^{\infty }‍{\mathrm{d\xi}}_{⊥}\frac{1}{\varepsilon_{\text{LRA}}^{2}\left(\xi_{⊥},V\right)}\frac{\partial }{\partial V}\varepsilon_{\text{LRA}}\left(\xi_{⊥},V\right)$$ (6a)

$$\frac{\partial d_{∥}}{\partial V}=\frac{1}{\varepsilon_{m}-\varepsilon_{d}}\int_{-\infty }^{\infty }‍{\mathrm{d\xi}}_{⊥}\frac{\partial }{\partial V}\varepsilon_{\text{LRA}}\left(\xi_{⊥},V\right)$$ (6b)

Here, ε_m_ and ε_d_ are the bulk permittivities of the metal and surrounding dielectric, respectively (for a detailed derivation, see section S1.5). For both components, the integrands in Eqs. 6a and 6b contain the partial derivative of the local permittivity with respect to the applied voltage ${\partial_{V^{\varepsilon }}}_{\text{LRA}}$. That is, they depend on the induced electron density Δ*n*_0_(ξ_⊥_, *V*) (see Eq. 5), which can be related to the classical electrostatic induced surface electron density Δη_0_(*V*) via the integral

$$\int_{-\infty }^{\infty }‍{\mathrm{d\xi}}_{⊥}\Delta n_{0}\left(\xi_{⊥},V\right)={\Delta \eta }_{0}\left(V\right)$$ (7)

The voltage-induced surface electron density can be obtained from the discontinuity of the normal component of the static displacement field *D*_⊥_ = ε_0_κ*E*_⊥_, where *E*_⊥_ is the normal component of the static electric field (see Fig. 3B) in the dielectric and κ is the corresponding dielectric constant. Furthermore, we can express the induced surface electron density in terms of a surface capacitance *C*_s_ as Δη_0_(*V*) ≃ *C*_s_*V*/*q*_e_, where *q*_e_ is the electron charge, showing explicitly the dependency on the applied voltage. This implies that we can represent the induced electron density as

$$\Delta n_{0}\left(\xi_{⊥},V\right)≃p\left(\xi_{⊥}\right)\frac{C_{s}V}{q_{e}}$$ (8)

where *p*(ξ_⊥_) is the probability distribution of the induced electron density per unit length, which follows the shape of the induced electron density (see Fig. 1B) and satisfies the normalization condition ∫⊥ *d*ξ_⊥_*p*(ξ_⊥_) = 1. In the case of the *d*_∥_ component, using Eq. 5 for the evaluation of Eq. 6b reduces to the evaluation of the partial derivative with respect to voltage of the integral in Eq. 7. Therefore, by using Eq. 8, the perturbation coefficient can be expressed analytically with a purely classical term

$$\frac{\partial d_{∥}\left(r_{s},V\right)}{\partial V}≃\frac{1}{\varepsilon_{d}-\varepsilon_{m}}\frac{\omega_{p}^{2}}{\omega^{2}+i\gamma \omega }\frac{C_{s}\left(r_{s}\right)}{q_{e}}\frac{1}{n_{0}}$$ (9)

which is independent of the shape of the induced electron density *p*(ξ_⊥_), with the resulting perturbation depending only on the amount of the induced charge Δ*d*_∥_(**r**_s_,*V*) ∝ *C*_s_(**r**_s_)*V*. Here, the surface position dependency **r**_s_ has been reintroduced through the surface capacitance *C*_s_(**r**_s_), arising from the uneven distribution of induced static charges. In this simple case when only the plasma frequency depends on the position (see Eq. 5), one can show that the perturbed *d*_∥_ parameter directly leads to a perturbed surface conductivity Δσ_s_ ∝ Δ*d*_∥_, the same result as provided by the classical model of Bohren and Hunt (for a detailed discussion see section S1.6). The phenomenological classical model can be derived as an approximation to our more general approach. In addition to the perturbation of the surface conductivity resulting from the bulk electrons, other nonclassical effects, such as conduction via surface states, can also affect the *d*_∥_ perturbation coefficient (*47*, *48*). This is especially relevant since our resonators are fabricated from single-crystalline gold microplatelets with Au(111) top and bottom surfaces (*54*, *55*).

In the case of the *d*_⊥_ component, by inserting Eq. 8 for the induced electron density into Eq. 5, the surface position dependency carried by the surface capacitance can be pulled outside the integral in Eq. 6a, which results in the following expression

$$\frac{\partial d_{⊥}\left(r_{s},V\right)}{\partial V}\approx \frac{\varepsilon_{d}\varepsilon_{m}}{\varepsilon_{d}-\varepsilon_{m}}\frac{\omega_{p}^{2}}{\omega^{2}+i\gamma \omega }\frac{C_{s}\left(r_{s}\right)}{q_{e}}\frac{1}{n_{0}}\int_{-\infty }^{\infty }‍{\mathrm{d\xi}}_{⊥}\frac{p\left(\xi_{⊥}\right)}{\varepsilon_{\text{LRA}}^{2}\left(\xi_{⊥}\right)}$$ (10)

Evaluation of the integral (Eq. 10) is not trivial since it depends on the shape of the induced electron density and the local permittivity. For simplicity, we assume that the background permittivity (Eq. 5) follows the distribution of the equilibrium electron density (see Fig. 3A), which is derived from DFT calculations using the jellium approximation. Subsequently, as one transitions from the metal to the dielectric, the equilibrium electron density smoothly changes from the bulk value to zero. This results in a position, ξ_⊥0_, where the local permittivity (real part) crosses zero. Therefore, accurately evaluating the integral in Eq. 10 necessitates a precise knowledge of the shape of the permittivity in the vicinity of ξ_⊥0_ (see section S1.7).

### Simulations

To evaluate the perturbation coefficients in Eqs. 9 and 10, we need to evaluate the equilibrium *n*_0_(ξ_⊥_), and the induced electron density Δ*n*_0_(ξ_⊥_, *V*), as well as the position-dependent surface capacitance *C*_s_(**r**_s_). To this end, we perform DFT calculations of a thin slab of jellium (8 nm) and electrostatic simulations for the geometries under investigation (for more details, refer to sections S1.7 and S1.8). In Fig. 3C, we show interface-averaged *d*-parameter perturbation coefficients ∂_*V*_*d*_i_(**r**_s_, ω) for the 140-nm-long resonator. Real and imaginary parts for all the perturbation coefficients exhibit negative values with magnitudes on the order of 0.1 pm/V and show small gradual variations across the spectrum. Therefore, for typical voltages of around 10 V, the resulting perturbations Δ*d*_i_(**r**_s_, ω) = ∂_*V*_*d*_i_(**r**_s_, ω)*V* will be approximately 1 pm. Negative signs in the real parts are expected, since for a positive bias, we deplete the surface from electrons, effectively reducing the size of the resonator. Although in Fig. 3C, we show only the case of the gold-glass interface, the result is similar for the gold-air interface (see section S1.9).

We can now investigate how the *d*-parameter perturbations ∂_*V*_*d*_i_(**r**_s_, ω)*V* induce a relative change in the scattering spectrum Δ*S*/*S*_0_(ω, *V*). Assuming that the change in scattering Δ_i_*S*(ω) scales linearly with the introduced perturbation Δ*d*_i_(**r**_s_) (see section S1.9), then each voltage-induced spectrally and spatially dependent perturbation ∂_*V*_*d*_i_(**r**_s_, ω)*V* will lead to a spectral change in scattering

$$\Delta_{i}S\left(\omega ,V\right)≃\frac{\Delta_{i}S\left(\omega \right)}{\Delta d_{i}\left(r_{s}\right)}\partial_{V}d_{i}\left(r_{s},\omega \right)V$$ (11)

As the spectral changes Δ_i_*S* are much smaller than the unperturbed scattering (Δ*S*_i_ << *S*_0_), we can treat each of them independently and calculate the total relative change as a linear combination of the individual contributions

$$\frac{\Delta S\left(\omega ,V\right)}{S_{0}}≃\sum_{i}‍\frac{1}{S_{0}}\frac{\Delta_{i}S\left(\omega \right)}{\Delta d_{i}\left(r_{s}\right)}\partial_{V}d_{i}\left(r_{s},\omega \right)V$$ (12)

As next step, we examine how each component of the *d*-parameters influences the scattering spectrum, i.e., we construct “basis functions” 1/*S*_0_ · Δ_i_*S*(ω)/Δ*d*_i_(**r**_s_). First, numerical simulations are conducted to obtain the scattering resonance as shown in the inset of Fig. 3D. The unperturbed resonance position ω_r0_ and width γ_r0_ are obtained by fitting a Lorentzian line shape multiplied with a linear background to the scattering spectrum *S*_0_ (see section S1.9). In the second step, we vary each of the interface-averaged *d*-parameters independently by 1 pm [Δ*d*_i_(**r**_s_) = 1 pm] and calculate the corresponding relative change of scattering Δ*S*_i_/*S*_0_(ω) using the mesoscopic boundary conditions (see Fig. 3D) (*40*). We find that the calculated changes are of the same order of magnitude as the experimentally observed ones, suggesting that we measure subpicometer shifts in the position of the optically induced electron density (see section S1.2). To characterize the impact of these changes on scattering amplitude and eigenfrequency, we fit each of the altered spectra using the relative change of the Lorentzian and retrieve Δ_i_*A*/*A*_0_, Δ_i_ω_r_, Δ_i_γ_r_ per unit perturbation Δ*d*_i_ as fitting parameters (for more details refer to section S1.9). We can see that for a real-part perturbation of 1 pm in the *d*_∥_(*d*_⊥_) component [denoted as Re_∥_(Re_⊥_) in Fig. 3D, respectively], we obtain dispersive curves with maximal changes on the slopes of the resonance, and a zero-crossing point almost perfectly aligned with the position of the resonance frequency. These characteristic shapes of the relative changes are predominantly produced by a change in the resonance position (compare to orange curves in Fig. 1E). Moreover, introducing a perturbation of 1 pm to the imaginary part of the *d*_∥_(*d*_⊥_) component [denoted as Im_∥_(Im_⊥_) in Fig. 3D] predominantly influences the width of the resonance (compare to green curve in Fig. 1E). We can see that the induced changes in scattering for two parameters are of similar magnitude but act in opposite directions, while the perturbation coefficients have the same sign, implying competing effects. This hints to a possible explanation why in quantum approaches dominated by a strong out-of-plane response it is expected to observe a red shift and resonance broadening for a negatively charged resonator (*28*, *29*). In contrast, classical models equivalent to the model proposed by Bohren and Hunt predict predominantly blue-shifted resonances, as the resonance is influenced by an in-plane response (*27*).

### Comparison of experiment and theory

**Fig. 4.**
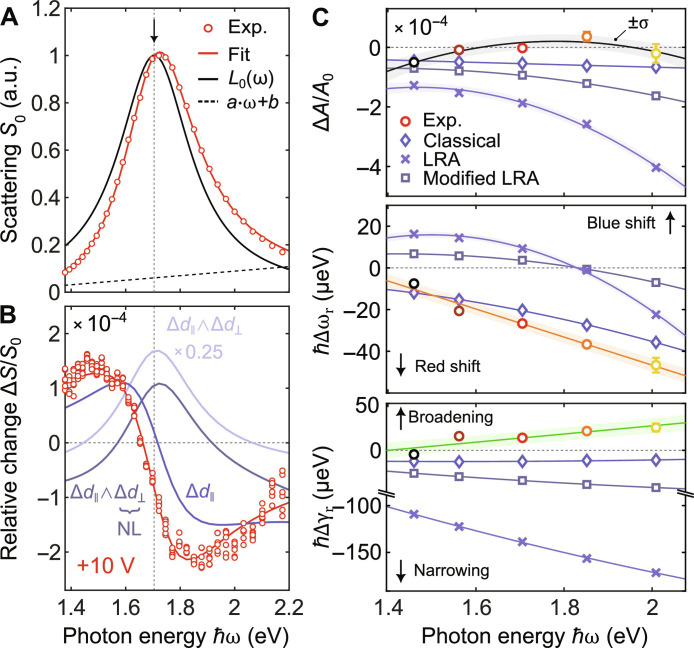
Comparison of experiment and theory. (**A**) Scattering spectrum for 140-nm-long resonator in experiment (red circles) and fitted curve (red curve); fitted curve is a product of a Lorentzian *L*_0_(ω) ≡ *L*(ω; *A*_0_, ω_r0_, γ_r0_) (black curve) and a line *a* · ω + *b* (black dashed line). (**B**) Relative change of scattering corresponding to resonances presented in (A) for a +10 V of applied voltage. Solid blue line corresponds to classical model, light-blue line to full LRA, and gray line to phenomenological nonlocality modified LRA. (**C**) Perturbations of resonance amplitudes, frequencies, and widths retrieved from experiments and theory for both classical (blue diamonds) and DFT models (light blue crosses and gray squares). Color code of the nanoresonators is identical to Fig. 2.

To ensure uniform treatment of theory and experiments and to simplify the analysis of experimental data, we first use Lorentzian fitting to the measured scattering resonance (see Fig. 4A). Subsequently, from the obtained resonance position and linewidth, together with the characteristic perturbation parameters describing the simulated basis functions, we construct the basis functions for our experimentally realized resonators. Then, by using Eq. 12, we calculate the total relative change in scattering for our experimentally realized resonators (for more details, refer to sections S1.9 and S1.10). In Fig. 4B, we present the experimental data alongside the relative change of scattering derived from theory for an applied potential of +10 V.

As a result, the perturbation caused solely by the *d*_∥_ component, which is identical to a purely classical treatment, exhibits a similar spectral shape as the experimental result. This resemblance is evident in the observed spectral shift ℏΔω_r_, which is approximately (−20.10 ± 0.09) μeV for the theoretical curve, while the measured value is (−26.7 ± 0.6) μeV; here, we use one SD to represent the uncertainties. However, a notable discrepancy arises when considering the relative change signal at the resonance frequency position, which can be attributed to a change in resonance width and/or amplitude (see Fig. 1D). We observe that the experimental resonance broadens by ℏΔγ_r_ = (13 ± 2) μeV with only a minimal change in amplitude Δ*A*/*A*_0_ = (−0.02 ± 0.08) × 10^−4^, whereas the theoretical resonance narrows ℏΔγ_r_ = (−12.2 ± 0.3) μeV and diminishes in amplitude Δ*A*/*A*_0_ = (−0.56 ± 0.01) × 10^−4^. When we include the *d*_⊥_ component perturbation, characterized by a substantial imaginary part (refer to Fig. 3C), we obtain a spectral curve resembling the shape of the unperturbed resonance, with a maximum of approximately 7 × 10^−4^ at the resonance frequency (see Fig. 4B). This spectral shape indicates a considerable resonance narrowing characterized by ℏΔγ_r_ = (−139.0 ± 0.1) μeV, accompanied by a small negative offset characterized with a relative change in amplitude Δ*A*/*A*_0_ of (−1.87 ± 0.01) × 10^−4^. Such characterization is visible from the shape of the resulting spectrum in Fig. 4B. In addition, we obtain a spectral shift of the opposite sign compared to the case when only the in-plane component perturbation ℏΔω_r_ = (−12.4 ± 0.4) μeV is considered.

## DISCUSSION

### Discussion of the relative contributions

Differences between experiment and classical approach for all three parameters that characterize a resonance perturbation show that taking only *d*_∥_ component perturbation is not sufficient. This becomes particularly evident for resonators of decreasing lengths, with the scattering spectra depicted in Fig. 2B, where discrepancies increase toward the blue end of the spectrum, as illustrated in Fig. 4C. To address these disagreements, the model needs to be extended by considering the nonclassical effects contained in the out-of-plane response. However, including the *d*_⊥_ component perturbation, derived from spill-out under LRA, considerably increases the deviation between theoretical results and experimental data, with the most notable difference observed in the change of linewidth induced by the pronounced imaginary part of the *d*_⊥_ component (see Fig. 4C). The absence of the *d*_⊥_-component effects, as calculated from DFT, suggests that contributions from the *d*_⊥_ component are suppressed in the experiment. Assuming that the DFT jellium calculations, at least qualitatively, capture the surface electrodynamic phenomena of the real physical surface, this leads to the conclusion that either nonlocal effects counteract the perturbation as calculated with the LRA and/or there is an anisotropy of the perturbed local permittivity with suppressed out-of-plane response. To investigate the impact of nonlocal effects, we follow approaches outlined in (*56*, *57*). By including a phenomenological nonlocal function into our analysis, we modify the shape of local permittivity, which leads to substantial changes in the *d*_⊥_ component perturbation (see section S1.11). Notably, we observe a considerable decrease in the imaginary part, which has a strong effect on the perturbation of the resonance linewidth (see Fig. 4B), ultimately reducing the discrepancy between the experimental data and modified theoretical model based on LRA (see Fig. 4C). Furthermore, a possible source of mismatch could be omission of the electron affinity of the dielectric and the near-field pseudopotential of the metal ion lattice in our DFT analysis, as discussed in (*29*, *58*). To investigate whether this is the source of disagreement, we inspect the spectral response of the system analyzed in (*29*) using our method based on the electron spill-out and modified LRA (see section S1.12). The resulting spectral changes are similar to those retrieved in the original study, indicating that electron affinity and near-field pseudopotential do not qualitatively impact the results. Nevertheless, we also show that for such a dimer antenna, even if only the in-plane component is perturbed, we can expect to observe a strong modulation of light scattering.

Last, if we assign all of the discrepancies between the experimental data and the classical *d*_∥_ component perturbation to nonclassical effects contained in the *d*_⊥_ component, we can leverage our analysis to estimate the *d*_⊥_ component perturbation. This involves conducting a fitting procedure on the spectral difference of the induced relative change in scattering, considering both experimentally observed values and the contributions from the in-plane components, across all nanoresonators. As a result of this analysis, we anticipate a small positive perturbation in the *d*_⊥_ component for both the real and imaginary parts in a complete contrast to calculated values (see section S2.6).

We have presented both theoretical and experimental investigations of charged plasmonic resonators and their optical response. In our experimental work, we have established direct electrical charging and measured the resulting change in the amplitude, resonance frequency, and linewidth. When applying a positive bias, the resonance shifts toward longer wavelengths while simultaneously increasing in width; the opposite effects occur under a negative bias. To explain the experimental observations, we analyze the charging effects within the general framework of surface response functions taking into account both in-plane and out-of-plane responses. Feibelman *d*-parameter perturbations are calculated from a basic model derived from the equilibrium and induced electron densities at a charged jellium-vacuum interface within the LRA. We have derived an analytical expression of the *d*_∥_ component perturbation and demonstrated that it exhibits a purely classical behavior, which is equivalent to a perturbation of the surface conductivity based on bulk parameters. Moreover, the perturbed in-plane response accounts for the large part of the experimentally observed spectral shift of the resonance frequency. However, discrepancies in all three parameters that characterize the change in resonance, especially for smaller resonators, indicate the presence of nonclassical effects contained in the out-of-plane response. Yet, including the out-of-plane response, based on the electron spill-out in LRA, considerably increases the deviation between theoretical results and experimental data indicating a strong resonance narrowing for a positively charged resonators. This is in complete contrast to experimentally obtained data, which suggest spectral narrowing as more electrons are added to the resonator. We attribute these discrepancies to unaccounted nonclassical effects that extend beyond the electron spill-out, such as nonlocality and anisotropy of the local permittivity. Already including the phenomenological nonlocality drastically changes the out-of-plane response and reduces the discrepancy between the experimental data and theoretical model based on LRA. Furthermore, using our analysis, we are able to estimate the size of the out-of-plane perturbation. Thus, to theoretically account for the out-of-plane response, further modeling efforts that go beyond jellium considerations are required. In addition, we observed that smaller resonators exhibit more pronounced changes in resonance behavior, because of their increased surface-to-volume ratio. If these resonators are further downsized, this effect is anticipated to become even more prominent, providing a possibility to detect the expected ultrafast modulation, fundamentally limited by the RC time constant of the system (*29*). Our experiment opens a vast field of investigations on how to gain control over the surface response in plasmonic resonators and to develop ultrafast and extremely small electrically driven plasmonic modulators and metasurfaces by leveraging electrical control over nonclassical surface effects.

## MATERIALS AND METHODS

### Numerical simulations

The perturbation of the optical response of a plasmonic nanoresonator under electrostatic biasing is numerically determined using the commercially available finite element method (FEM) solver (COMSOL Multiphysics 6.0) (*59*) for the electrodynamics. Our simulations involve a two-step process. Initially, we solve for the electrostatic field by applying a potential *V* to the structure using the AC/DC module. This step allows us to obtain the induced surface electron density Δη_0_(**r**_s_, *V*). The ground potential is placed infinitely far away by using an infinite element domain layer. In the second step, we conduct optical simulations using the wave optics module to analyze the scattering cross section based on the local perturbation in the *d*-parameters influenced by the induced surface electron density. To introduce perturbations in the *d*-parameters, we use mesoscopic boundary conditions (see section S1.3) implemented with an auxiliary potential method, as described in (*40*). The structure is excited with a plane wave polarized along the long axis of the nanoresonator, and the scattered light is collected at the bottom hemisphere to mimic the experimental setup. The optical permittivity of gold is taken from the experimental values for monocrystalline gold provided by Olmon *et al.* (*60*), while glass is modeled using Sellmeier coefficients. For more information, please refer to section S1.8.

### Sample fabrication

Monocrystalline gold microplatelets, measuring 50 nm in thickness, are synthesized through a wet chemical process outlined in (*52*). These microplatelets are then transferred onto a glass coverslip (24 mm × 24 mm, #1.5 Menzel) with evaporated metal layers featuring an array of electrode pads prepared by optical lithography and electron beam physical vapor deposition (20-nm chromium adhesion layer and 80-nm gold layer). The microplatelets are carefully positioned over the glass window on structured microscopic electrodes, ensuring a conductive connection between the flake and the metal film. Nanoresonator fabrication is conducted as described in (*53*).

### Optical characterization

To capture dark-field scattering spectra of plasmonic nanoresonators, we use an inverted optical microscope (TE2000-U, Nikon) equipped with a nanopositioning piezostage (NanoLPS200, Mad City Labs Inc.) and an oil immersion microscope objective (PlanApochromat, 100×, numerical aperture = 1.45, Nikon). As excitation source, we use a supercontinuum laser (SuperK FIANIUM, FIR-20, NKT) that is spectrally shaped and scanned in 10 nm increments from 500 to 900 nm using an acousto-optic tunable filter (SuperK SELECT, NKT). The light from the laser is sent through a 300-μm pinhole and collimated via a 500-mm lens, providing a weakly focused beam at the sample. To separate the detection and excitation beam paths, a 50:50 beam splitter is used. Light scattered by the structure above the critical angle and light reflected directly from the sample are separated using a circular beam block. In addition, to minimize potential stray light, an iris is positioned in the intermediate image plane and adjusted until the background is completely suppressed. The signal is collected using an optical power meter (1835-C, Newport). See also sections S2.1 to S2.3.

### Electro-optical measurements

The nanoresonators are electrically connected to the macroscopic electrode pads using thin connector lines. They are then further connected via micromanipulators to a home-built voltage amplifier driven by a function generator (DS 345, Standford Research Instruments). An AC voltage is applied to the structure, and the scattered light, guided to the detector, is divided into two collecting channels using a 90:10 beam splitter. The majority of the light is captured by a silicon photodetector (DET36A2, Thorlabs) with a rise time of 14 ns, while the remaining portion is detected using an optical power meter (1835-C, Newport). The electrical signal from the photodetector is directed to a lock-in amplifier (DSP 7260, EG&G), with the reference signal obtained directly from the function generator output using a T-splitter. To enable the recording of correlated data, the entire process is monitored via a LabVIEW program. Throughout the measurements, the sample is continuously blow-dried using a laminar nitrogen stream.

## Supplementary Materials

### This PDF file includes:

Sections S1 and S2 Figs. S1 to S15 References
