# Supplementary material for: Modulation of surface response in a single plasmonic nanoresonator
Source: Sci Adv. 2024 Sep 6;10(36):eadn5227. doi: 10.1126/sciadv.adn5227 (PMC11378946; doi:10.1126/sciadv.adn5227)
Supplement: Supplementary file 1 — Sections S1 and S2 Figs. S1 to S15 References [file sciadv.adn5227_sm.pdf]

Science Advances
AAAS

Supplementary Materials for

Modulation of surface response in a single plasmonic nanoresonator

Luka Zurak *et al.*

Corresponding author: Luka Zurak, luka.zurak@uni-wuerzburg.de; Thorsten Feichtner, thorsten.feichtner@uni-wuerzburg.de

Sci. Adv. **10**, eadn5227 (2024)
DOI: 10.1126/sciadv.adn5227

This PDF file includes:

Sections S1 and S2
Figs. S1 to S15
References

1 Model

1.1 Classical models

When discussing the optical response of charged plasmonic nanoresonators, two classical models are relevant. These models assume that the excess charge arises from a variation in the number of free electrons N_0.

Bulk model

In a simplified yet illustrative scenario, an excess charge is uniformly distributed throughout the volume V of the resonator – one should note that due to screening this assumption is obviously incorrect and misleading. Despite it's flaws, this model is quite commonly employed to provide a rough estimate of the magnitude of the induced change. Consequently, this distribution induces changes in the material properties of the resonator by altering the bulk conductivity [61]:

$$\sigma_\mathrm{b} = i\varepsilon_0 \frac{\omega_\mathrm{p}^2}{\omega + i\gamma}, \tag{S1}$$

where $\omega_\mathrm{p} \propto \sqrt{n_0}$ is the plasma frequency and $n_0 = N_0/V$ is the free electron density. It can be shown that the resonance frequency of the system is proportional to the plasma frequency of the system. Therefore, changing the density of the free electrons effectively causes a change in the resonance frequency.

Surface model

Bohren and Hunt introduced a surface current model [27], where the excess charge induces a change in the surface electron density η_0, resulting in a modification of the surface conductivity σ_s. By applying the Drude model to the thin surface charge layer consisting of electrons with mass m and charge q_e, the surface conductivity can be expressed using the following equation:

$$\sigma_\mathrm{s} = i\frac{q_\mathrm{e}^2}{m}\frac{\eta_0}{\omega + i\gamma}, \tag{S2}$$

where γ is the collision rate at the surface. When the surface conductivity is perturbed, it induces a modification in the surface current $\mathbf{K} = \sigma_\mathrm{s}\mathbf{E}_{||}$, where $\mathbf{E}_{||}$ is the tangential component of the electric field at the surface. To incorporate the presence of the surface current in Maxwell's equations, the boundary condition for the tangential component of the magnetic field $\mathbf{H}$ needs to be redefined as

$$\Delta\mathbf{H}_{||} = \mathbf{K} \times \hat{\xi}_\perp, \tag{S3}$$

where $\hat{\xi}_\perp$ is the surface normal unit vector pointing from material 1 to material 2 and $\Delta\mathbf{H} = (\mathbf{H}_2 - \mathbf{H}_1)$. The implications of the surface model, regarding its influence on the resonance, are not as clear as those in the case of the bulk model. However, a direct comparison can be made when discussing the special case of a sphere [27]. For spheres with very small radius r compared to the wavelength λ of light in the surrounding medium ($r \ll \lambda = 2\pi/k$), the only relevant coefficient using Mie expansion is the first one. Therefore, one can express the extinction cross section as

$$\sigma_\mathrm{ext} = 4kr\,\mathrm{Im}\left[\frac{\varepsilon_\mathrm{eff} - 1}{\varepsilon_\mathrm{eff} + 2}\right] \tag{S4}$$

where ε_eff is the effective relative permittivity of the particle, with the resonance condition reached for the frequency where $\mathrm{Re}(\varepsilon_\mathrm{eff}) = -2$. The effective relative permittivity is given as a sum of bulk and surface terms:

$$\varepsilon_\mathrm{eff} = \varepsilon_\mathrm{b} - i\frac{\sigma_\mathrm{b} + 2\sigma_\mathrm{s}/r}{\omega}, \tag{S5}$$

Here ε_b is the background permittivity due to interband transitions. This equation implies that the surface conductivity contributes to the bulk conductivity as $2\sigma_\mathrm{s}/r$, or in other words, the relative change of the resonance is now proportional to the relative change of the effective plasma frequency, containing the surface term

$$\omega_\mathrm{p,eff} \propto \left(n_0 + 2\frac{\eta_0}{r}\right)^{1/2}. \tag{S6}$$

Therefore, for very small systems the surface model should provide qualitatively similar result as the bulk, leading to the perturbation of the resonance frequency.

1.2 Feibelman d-parameters

In classical electrodynamics, an interface between a metal and a dielectric is considered to be infinitesimally thin, suggesting that the induced charge density $\rho(\xi_\perp)$ is strictly confined to the interface, meaning $\rho(\xi_\perp) \propto \delta(\xi_\perp)$ [49], where $\delta(\xi_\perp)$ is the Dirac delta function and $\xi_\perp$ is the spacial axis perpendicular from the surface. However, in his seminal work [30], Feibelman demonstrated that the first-order correction to the multipole expansion of the induced charge density introduces two additional quantities known as "d-parameters". The d-parameters, denoted as $d_\perp \equiv d_\perp(\omega)$ and $d_{||} \equiv d_{||}(\omega)$, are frequency-dependent centroids of the induced charge density, and of the normal derivative of the tangential current $\mathbf{J}_{||}(\xi_\perp)$, respectively (see Ref. [49]). That is, we can express them with the following equations:

$$\begin{aligned} d_\perp &= \frac{\int_{-\infty}^{\infty} \xi_\perp \rho(\xi_\perp) d\xi_\perp}{\int_{-\infty}^{\infty} \rho(\xi_\perp) d\xi_\perp}, \\ d_{||} &= \frac{\int_{-\infty}^{\infty} \xi_\perp \frac{\partial}{\partial \xi_\perp} J_{||}(\xi_\perp) d\xi_\perp}{\int_{-\infty}^{\infty} \frac{\partial}{\partial \xi_\perp} J_{||}(\xi_\perp) d\xi_\perp}. \end{aligned} \tag{S7}$$

The d-parameters serve as descriptors of the optical properties of realistic metal-dielectric interfaces, similar to how the permittivity characterizes the bulk properties. They provide information about the nonlocality and inhomogeneous nature of the medium in proximity of the interface. Consequently, they are an appropriate tool for investigating the impact of electrostatically induced surface charge on the optical properties of plasmonic nanoresonators.

Typically, the d-parameters are obtained through ab-initio calculations that examine the response of metal surfaces to time-varying electric fields. For example, time-dependent density-functional theory (TD-DFT) is commonly employed for this purpose [62, 63]. In certain cases, analytical evaluations of the response can be performed, using e.g. semiclassical hydrodynamic models [64-66]. However, in all instances, the common approach to retrieve the d-parameters has been to assess the non-equilibrium electron response and employ equations (S7).

1.3 Mesoscopic boundary conditions

The d-parameters can be introduced into classical equations by means of quantum-corrected boundary conditions [40], as they govern the surface polarization given with Eq. (1) in the main text. When incorporating this additional surface polarization at the interface between two materials, the revised boundary conditions are as follows:

$$\Delta D_\perp = -i\omega^{-1} \nabla_{||} \cdot \mathbf{K} = d_{||} \nabla_{||} \Delta \mathbf{D}_{||} \tag{S8a}$$

$$\Delta B_\perp = 0 \tag{S8b}$$

$$\Delta \mathbf{E}_{||} = -\frac{1}{\varepsilon_0} \nabla_{||} \pi = -d_\perp \nabla_{||} \Delta E_\perp \tag{S8c}$$

$$\Delta \mathbf{H}_{||} = \mathbf{K} \times \mathbf{n} = i\omega d_{||} \Delta \mathbf{D}_{||} \times \mathbf{n} \tag{S8d}$$

Boundary conditions are implemented in FEM simulations using the auxiliary-potential method as discussed in Ref. [40].

1.4 The lossy harmonic oscillator

In classical terms, the resonance of a lossy harmonic oscillator can be characterized using a Lorentzian function $L(\omega; A, \omega_\mathrm{r}, \gamma_\mathrm{r})$. This function is defined by parameters including the amplitude A, resonance position ω_r, and width γ_r (see Fig. 1D in the main text), expressed as follows:

$$L(\omega; A, \omega_\mathrm{r}, \gamma_\mathrm{r}) = \frac{A}{(\omega - \omega_\mathrm{r})^2 + \gamma_\mathrm{r}^2/4} \tag{S9}$$

A small change in amplitude, denoted by ΔA, induces a constant relative change across the spectrum (refer to Fig. 1E in the main text). This can be analytically computed by considering the first-order term in the Taylor

expansion of the Lorentzian function, which is expressed as follows:

$$\frac{\Delta L_A}{L_0} \simeq \frac{1}{L_0}\frac{\partial L}{\partial A}\bigg|_0 \Delta A = \frac{\Delta A}{A_0} \tag{S10}$$

where $\Delta L_A \equiv L(\omega; A_0 + \Delta A, \omega_{r0}, \gamma_{r0},) - L_0$ and $L_0 \equiv L(\omega; A_0, \omega_{r0}, \gamma_{r0})$ is the unperturbed resonance, described with amplitude A_0, resonance position ω_{r0} and width γ_{r0}.

Similarly, a small perturbation in the resonance frequency, denoted by $\Delta\omega_r$, results in a unique spectral shape representing the relative change in resonance, as illustrated in Fig. 1E of the main text. This spectral shape is described by the following expression:

$$\frac{\Delta L_{\omega_r}}{L_0} \simeq \frac{1}{L_0}\frac{\partial L}{\partial \omega_r}\bigg|_0 \Delta\omega_r = \frac{2L_0}{A_0}(\omega - \omega_{r0})\Delta\omega_r\,, \tag{S11}$$

where $\Delta L_{\omega_r} \equiv L(\omega; A_0, \omega_{r0} + \Delta\omega_r, \gamma_{r0}) - L_0$. Extrema of the relative change curve are reached at frequencies $\omega_\pm = \omega_{r0} \pm \gamma_{r0}/2$, where the relative change of resonance is

$$\frac{\Delta L_{\omega_r}}{L_0}\bigg|_{\omega=\omega_\pm} = \pm 2Q_0 \frac{\Delta\omega_r}{\omega_{r0}}\,. \tag{S12}$$

Here, $Q_0 = \omega_{r0}/\gamma_{r0}$ stands for the quality factor of the unperturbed resonance. If the resonance experiences a blueshift ($\Delta\omega_r > 0$), the relative change peaks on the blue side of the resonance and dips to a minimum on the red side. Conversely, in the case of a redshifted resonance ($\Delta\omega_r < 0$), we anticipate observing the inverse pattern with respect to the ordinate. Therefore, the change in resonance position can be deduced by analyzing the slope of the relative change curve at the resonance frequency, given with the following expression:

$$\frac{\partial}{\partial\omega}\frac{\Delta L_{\omega_r}}{L_0}\bigg|_{\omega=\omega_{r0}} = \frac{8\Delta\omega_r}{\gamma_{r0}^2} = \frac{8Q_0}{\gamma_{r0}}\frac{\Delta\omega_r}{\omega_{r0}}\,. \tag{S13}$$

To assess the magnitude of higher-order contributions, we can examine the pure quadratic term in the Taylor expansion, expressed as follows:

$$\frac{1}{2}\frac{1}{L_0}\frac{\partial^2 L}{\partial\omega_r^2}\bigg|_0 \Delta\omega_r^2 = \left[\frac{4L_0^2}{A_0^2}(\omega - \omega_{r0})^2 - \frac{L_0}{A_0}\right]\Delta\omega_r^2 \tag{S14}$$

The first term on the right side becomes maximal at $\omega_\pm = \omega_{r0} \pm \gamma_{r0}/2$, which is equal to the square of the expression in equation (S12). Likewise, the absolute maximum of the second term is of the same magnitude and is reached at $\omega = \omega_{r0}$. Given our expectation for the relative change of a resonance frequency to be on the order of 10^{-5}, both of these terms are negligible.

Moreover, a slight perturbation in the resonance width, denoted by $\Delta\gamma_r$, will also result in a characteristic spectral shape of the relative change curve (refer to Fig. 1E in the main text). This line shape is described by the following expression:

$$\frac{\Delta L_{\gamma_r}}{L_0} \simeq \frac{1}{L_0}\frac{\partial L}{\partial \gamma_r}\bigg|_0 \Delta\gamma_r = -\frac{L_0}{A_0}\frac{\gamma_{r0}}{2}\Delta\gamma_r\,, \tag{S15}$$

where $\Delta L_{\gamma_r} \equiv L(\omega; A_0, \omega_{r0}, \gamma_{r0} + \Delta\gamma_r) - L_0$. Thus, the relative change of line shape follows the shape of the resonance, and an extremum is reached at the resonance frequency ω_{r0}, with a value of:

$$\frac{\Delta L_{\gamma_r}}{L_0}\bigg|_{\omega=\omega_{r0}} = -2\frac{\Delta\gamma_r}{\gamma_{r0}}, \tag{S16}$$

where an increase/decrease in damping leads to an increase/decrease in resonance width and therefore to a negative/positive relative change.

The total relative change is simply a sum of contributions given with Eqs. (S10), (S11), and (S15)

$$\frac{\Delta L}{L_0} = \frac{\Delta L_A}{L_0} + \frac{\Delta L_{\omega_r}}{L_0} + \frac{\Delta L_{\gamma_r}}{L_0} = \frac{\Delta A}{A_0} + \frac{L_0}{A_0}\left[2(\omega - \omega_{r0})\Delta\omega_r - \frac{\gamma_{r0}}{2}\Delta\gamma_r\right]. \tag{S17}$$

1.5 Electron spill-out and LRA

A recent study [42] has revealed that even when only considering the spill-out effect and employing the LRA, there remains a finite contribution to the metallic surface-response functions. They can be obtained by considering the equilibrium electron density, assuming a smooth transition from its bulk value deep within the metal to zero outside. Calculating these contributions involves evaluating the following expressions

$$d_\perp = \frac{1}{\varepsilon_\mathrm{m}^{-1} - \varepsilon_\mathrm{d}^{-1}} \int_{-\infty}^{\infty} d\xi_\perp \left[\varepsilon_\mathrm{LRA}^{-1}(\xi_\perp) - \varepsilon_\mathrm{PCA}^{-1}(\xi_\perp)\right], \tag{S18a}$$

$$d_\parallel = \frac{1}{\varepsilon_\mathrm{m} - \varepsilon_\mathrm{d}} \int_{-\infty}^{\infty} d\xi_\perp \left[\varepsilon_\mathrm{LRA}(\xi_\perp) - \varepsilon_\mathrm{PCA}(\xi_\perp)\right], \tag{S18b}$$

which incorporate the dielectric function of the surrounding materials $\varepsilon_\mathrm{m}, \varepsilon_\mathrm{d}$ and its profile near the interface. Here, $\varepsilon_\mathrm{LRA}(\xi_\perp)$ is the smoothly varying dielectric function governed by the electron spill-out (as described with Eq. (4) in the main text), while $\varepsilon_\mathrm{PCA}(\xi_\perp)$ is the commonly assumed piecewise-constant dielectric function.

Since the dielectric function in the LRA depends on the equilibrium electron density, for an applied voltage V the electron density will be perturbed at the interface with the surrounding dielectric material, resulting in a new equilibrium electron density $n_0(\xi_\perp, V) = n_0(\xi_\perp) + \Delta n_0(\xi_\perp, V)$. Consequently, this leads to the perturbation of the local dielectric function $\varepsilon_\mathrm{LRA}(\xi_\perp, V) = \varepsilon_\mathrm{LRA}(\xi_\perp) + \Delta\varepsilon_\mathrm{LRA}(\xi_\perp, V)$. To inspect the influence of the applied voltage on the d-parameters (see Eq. (3) in the main text) we need to calculate the partial derivatives of the expressions given by equations (S18a) and (S18b):

$$\frac{\partial d_\perp}{\partial V} = \frac{1}{\varepsilon_\mathrm{m}^{-1} - \varepsilon_\mathrm{d}^{-1}} \int_{-\infty}^{\infty} d\xi_\perp \frac{\partial}{\partial V} \varepsilon_\mathrm{LRA}^{-1}(\xi_\perp, V), \tag{S19a}$$

$$\frac{\partial d_\parallel}{\partial V} = \frac{1}{\varepsilon_\mathrm{m} - \varepsilon_\mathrm{d}} \int_{-\infty}^{\infty} d\xi_\perp \frac{\partial}{\partial V} \varepsilon_\mathrm{LRA}(\xi_\perp, V). \tag{S19b}$$

From here the equations (6a) and (5b) in the main text follow immediately.

1.6 Surface-response functions and classical surface model

If we make the assumption that the perturbation caused by the $d_\perp$ component on the parallel component of the electric field is negligible (from equation (S8c) $\Delta\mathbf{E}_\parallel \approx \mathbf{0}$), then we can approximate $\Delta\mathbf{D}_\parallel$ as $\varepsilon_0(\varepsilon_\mathrm{d} - \varepsilon_\mathrm{m})\mathbf{E}_\parallel$. Consequently, the expression for the perturbation of the surface current is

$$\Delta\mathbf{K} = i\omega\Delta d_\parallel \Delta\mathbf{D}_\parallel \approx i\omega\varepsilon_0(\varepsilon_\mathrm{d} - \varepsilon_m)\Delta d_\parallel \mathbf{E}_\parallel. \tag{S20}$$

If we insert the $d_\parallel$ component perturbation $\Delta d_\parallel$ from the main text, we obtain

$$\Delta\mathbf{K} \approx i\varepsilon_0 \frac{\omega_\mathrm{p}^2}{\omega + i\gamma} \frac{\Delta\eta_0}{n_0} \mathbf{E}_\parallel = \Delta\sigma_\mathrm{s}\mathbf{E}_\parallel \tag{S21}$$

This result is fully equivalent to one obtained from the classical surface model introduced by Bohren and Hunt [27].

1.7 Evaluating the d-parameters

Equations (6a) and (10) in the main text involve an integral where the integrand depends on the inverse square of the local permittivity and the shape of the induced electron density. In order to address this, we conduct density functional theory (DFT) calculations using the "Jellium" approximation, which allows us to obtain the equilibrium electron densities as depicted in Fig. S1a. Specifically, we present the results for the case of $r_\mathrm{s} = 3.18\, a_0$ (a_0 is the Bohr radius) corresponding to a bulk electron density of $n_0 \approx 5 \cdot 10^{28}\,\mathrm{m}^{-3}$, determined from a Drude model fit to the material data as discussed in SM section 1.8. To evaluate the integral, it is

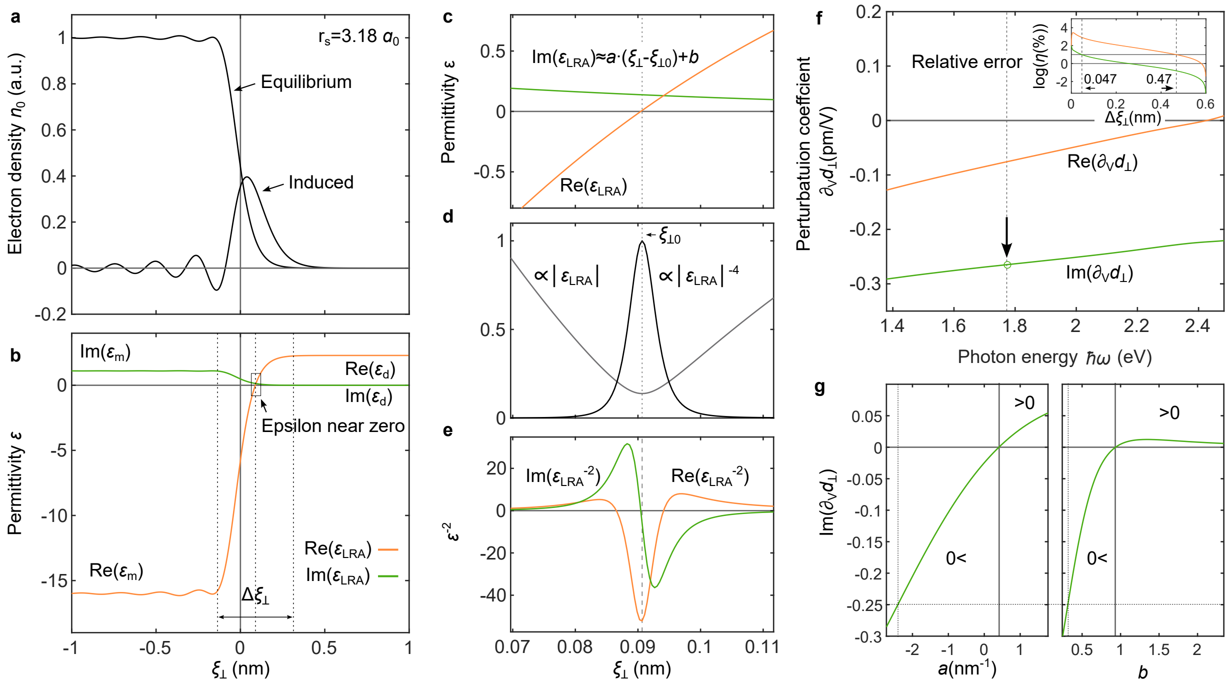

FIG. S1: **Evaluating the $d_\perp$ perturbation coefficient.** **a**, Equilibrium and induced electron density for r_s/a_0 =3.18. **b**, Spatial dependency of local permittivity on gold-glass interface. **c**, Zoom-in region where local permittivity approaches zero denoted with black rectangle in panel b. **d**, Plot of absolute value of local permittivity and inverse of fourth power in epsilon near zero region. **e**, Real and imaginary part of square of inverse local permittivity in epsilon near zero region. **f**, Spectrally depdendent interface-averaged perturbation coefficient of the $d_\perp$ component. **g**, Influence of the imaginary part of local permittivity represented with line on the imaginary part of the perturbation coefficient with change introduced to line slope (left) and line y-intercept (right).

necessary to choose a spatially varying background permittivity $\varepsilon_b(\xi_\perp)$. Deep inside the metal, it corresponds to the interband transitions contribution, $\varepsilon_{bm} = \varepsilon_m - \varepsilon_D$, where ε_D represents the Drude free-electron contribution. In the dielectric, it corresponds to the material permittivity ε_d. We adopt a smooth function that connects these two contributions, following the equilibrium electron density

$$\varepsilon_b(\xi_\perp) = n_0(\xi_\perp) \cdot \varepsilon_{bm} + [1 - n_0(\xi_\perp)] \cdot \varepsilon_d \tag{S22}$$

The resulting smooth local permittivity ε_{LRA} is illustrated in Fig. S1b. Due to the metal's significantly negative real part of permittivity and a small positive real permittivity of the dielectric, there exists a location where the real part of the local permittivity crosses zero. This specific region, which is denoted with a black rectangle in Fig. S1b, is significant for the integral evaluation since the integrand contains the inverse square of the local permittivity, ε_{LRA}^{-2}, which we can restate as follows:

$$\frac{1}{\varepsilon_{LRA}^2} = \frac{(\varepsilon_{LRA}^*)^2}{|\varepsilon_{LRA}|^4}. \tag{S23}$$

In Fig. S1c a zoomed-in view of the real and imaginary part of the local permittivity is shown in the vicinity where epsilon approaches zero. Additionally, we show the absolute value $|\varepsilon_{LRA}|$ in Fig. S1d. It is evident that the absolute value of local permittivity exhibits an asymmetry with respect to the position of the minimum, denoted as $\xi_{\perp 0}$. Specifically, the values on the left side ($\xi_\perp < \xi_{\perp 0}$) are slightly larger than the values at equidistant locations on the right side ($\xi_\perp > \xi_{\perp 0}$). As a result, $|\varepsilon_{LRA}|^{-4}$ will exhibit a contrasting asymmetry, enhancing contributions from the right side of the peak position. Consequently, both the real and imaginary parts of the integrand, ε_{LRA}^{-2}, will exhibit the same asymmetry, as depicted in Fig. S1e. From this we determine the perturbation coefficient for the gold-glass interface as presented in Fig. S1f. In the inset of the figure, we

provide a logarithmic plot of the absolute relative error determined using the following expression:

$$\eta(\Delta\xi_\perp) = \frac{\left|\partial_V d(\Delta\xi_\perp) - \partial_V d\right|}{\left|\partial_V d\right|}. \tag{S24}$$

It showcases the dependence of the real and imaginary parts of the perturbation coefficient on the size of the integration interval $\Delta\xi_\perp$ (illustrated in Fig. S1b), centered around the location of minimum $\xi_{\perp 0}$. It is apparent that in order to accurately determine the real value with a relative error of 10% or less, a relatively large integration interval, larger than 0.5 nm is required. On the other hand, the same error for the imaginary part can be achieved with an integration interval of approximately $\Delta\xi_\perp \approx 0.05$ nm. Therefore, the shape of the local permittivity within a relatively small spatial interval plays a crucial role in determining the imaginary part of the perturbation coefficient. To investigate this influence, we maintain the real part of the permittivity $\mathrm{Re}(\varepsilon_{\mathrm{LRA}})$ as given by the model and assume that the imaginary part can be approximated by a linear equation, $\mathrm{Im}(\varepsilon_{\mathrm{LRA}}) \approx a(\xi_\perp - \xi_{\perp\mathrm{m}}) + b$. In Fig. S1g, we illustrate the impact of the line slope, a (left), and the y-intercept, b (right), on the imaginary part of the perturbation coefficient. It can be observed that by increasing the line slope in a positive manner, the perturbation coefficient can be enhanced and rendered positive for $a > 0.3\ \mathrm{nm}^{-1}$. Similarly, a positive perturbation coefficient can be achieved by increasing the y-intercept past the value of 0.9. This can be understood as introducing a change in asymmetry to the absolute value of local permittivity which is then translated to real and imaginary part of the inverse square of the permittivity.

1.8 Nanoresonator on a substrate

When dealing with a realistic geometry, such as a rectangular nanoresonator on a glass substrate (as depicted in Fig. S2a), the distribution of the induced surface charge $\Delta\eta_0(\mathbf{r}_s, V)$ is non-uniform. In order to determine the response of such a system, we utilize finite element method (FEM) simulations and solve Maxwell's equations in a two-step procedure using a commercially available numerical solver (COMSOL Multiphysics). In the initial step, we solve for the static electric field and retrieve the induced surface charge density. Subsequently, we conduct simulations at optical frequencies. The simulation volume consists of a sphere with a radius r_{sur} of 400 nm and a spherical shell with a thickness t_{a} of 200 nm (see Fig. S2a). This layer serves as the infinite element domain (IED) for electrostatic simulations and the perfectly matched layer (PML) for optical simulations. In Fig. S2b, we present the geometry of the nanoresonator, which closely resembles the structures realized in the experimental setup. The nanoresonator's dimensions are as follows: length $l = 140$ nm, width $w = 80$ nm, and height $h = 50$ nm. Radius of curvature is $r = 8$ nm. Due to the fabrication procedure, it is surrounded by a trench with a depth $t_{\mathrm{d}} = 40$ nm and a width $t_{\mathrm{w}} = 50$ nm (similar to Ref. [53]. Due to the rough prestructuring with Ga-FIB, it stands atop a glass pedestal with a total height $p = 50$ nm. The single resonator exhibits a dipolar mode, as evidenced by the simulated radiation pattern (see inset of Fig. S2c). The peak position occurs around 1.7 eV, and the FWHM is roughly 280 meV. As in the experiment we solely observe light scattered above the critical angle, we conducted calculations to confirm that the shape of the resonance remains unaffected by the measurement procedure. To this end we compared the scattered power above the critical angle to the total scattered power (see Fig. S2c). In the case of electrostatic simulations, the surface of the nanoresonator is assumed to be an equipotential, with an electric potential V, while a reference potential V_{ref} (ground) is positioned at an infinitely far distance by employing the IED. The dielectric constant of the glass κ_{glass} is set to 5, which falls within the typical range for glasses (from 5 to 10). This enhances the capacitance at the interface between gold and glass. At the corners of the nanoresonator, the static electric field reaches values of 0.35 GV/m, while the average induced surface charge density amounts to 2.7 mC/m^2. By integrating the induced charge density over the nanoresonator's surface for an applied potential of 10 V, the total induced charge is approximately 0.1 fC. This charge is equivalent to removing approximately 700 electrons from the nanoresonator's surface, resulting in a total self-capacitance of the system of around 10 aF. For our optical simulations, we use the Olmon SC material data for gold, which we fit using a plasma frequency of $\hbar\omega_{\mathrm{p}} = 8.3$ eV and collision time of $\tau = 14$ fs. The considered spectral range spans from 0.5 eV (2500 nm) to 1.24 eV (1000 nm), where the free electron response dominates. Due to the dependence of the plasma frequency on both the free electron density and effective mass, it is not possible to obtain the exact value for the unperturbed bulk density n_0 of the system. However, assuming that the effective mass of the electron is equal to the rest mass of an electron $m = m_{\mathrm{e}}$, the unperturbed free electron density is $n_0 \approx 5 \cdot 10^{28}\mathrm{m}^{-3}$. To examine the impact of the additional electrons, we apply both

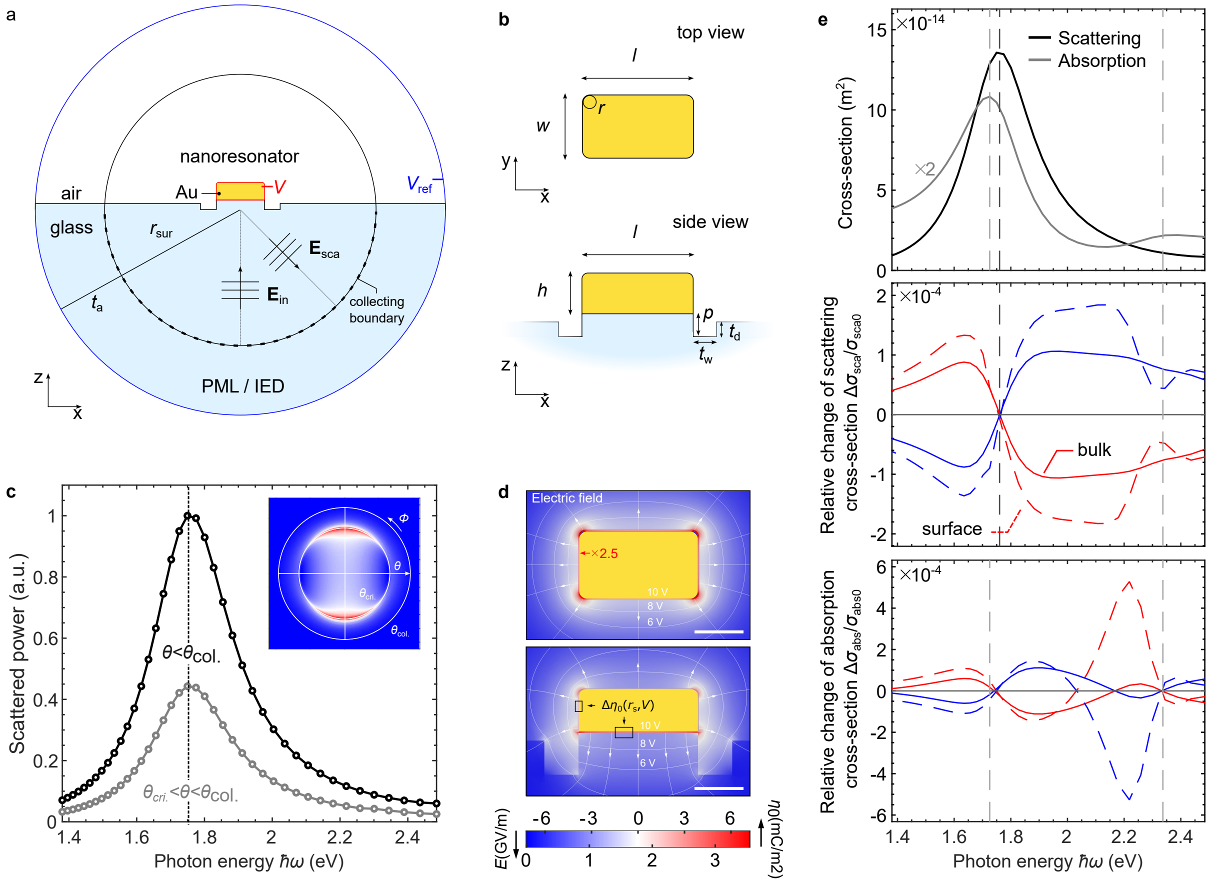

FIG. S2: **Electrically charged nanoresonator on a glass substrate.** **a**, Side view of the complete simulation geometry. In the electrostatic simulations, surface of the structure is an equipotential on a potential V, while the reference potential V_{ref}, situated on the outer surface of the IED, is set to zero volts. The structure is excited from below at a zero angle, using a plane wave characterized by the electric field $\mathbf{E}_{\text{in}}$. The light scattered by the structure is carried by the scattered electric field $\mathbf{E}_{\text{sca}}$, collected at the bottom hemisphere. **b**, Geometry of the nanoresonator with parameters (details in main text). **c**, The simulated scattering spectra are obtained by integrating the scattered power over specific regions of the hemisphere through radiation pattern calculations. The inset illustrates a radiation pattern. **d**, Results of electrostatic simulations. Electrostatic field and induced surface electron density as emphasized using black rectangles. **e**, (top) The absorption and scattering cross-section are presented. (middle) The relative change of the scattering cross-section. (bottom) The relative change in absorption cross-section.

classical models. In the surface model, we introduce a surface current $\mathbf{K}$, with conductivity determined from equation (S2). This model is implemented in the numerical solver through the application of built-in boundary conditions for the tangential component of the magnetic field, as stated with equation (S3). The results for both models are illustrated in Fig. S2e. We observe a similar behavior in the relative change of scattering for both the bulk and the surface model, reaching values of approximately 1×10^{-4} on the resonance slopes, with no alteration at the resonance frequency. This pattern of the relative change is characteristic of a resonance frequency shift (see Fig. 1e in the main text). Although the most significant change occurs near the resonance frequency, there are additional undulations in the relative change at around 2.2-2.3 eV. These changes are attributed to the presence of a dark mode, which is not clearly discernible in the scattering cross-section but is observable in the absorption cross-section.

1.9 Effect of the d-parameters on the properties of the plasmonic resonance

A small perturbation in one of the d-parameters $\Delta d_i(\mathbf{r}_s) \propto C_s(\mathbf{r}_s)$, where $i = \{\perp, \parallel\}$, will induce a change in the scattering cross-section $\Delta_i\sigma_{\text{sca}}$ i.e. scattering $\Delta_i S$. If we assume that each of the induced changes is smaller

than the unperturbed scattering $\Delta_i S \ll S_0$, we can treat them independently. Therefore, the total change of scattering is given as a sum of the independent contributions:

$$\Delta S \approx \sum_i \Delta_i S \,. \tag{S25}$$

Here, the assumption is that the individual changes scale linearly with the perturbation. We demonstrate linearity by calculating change in scattering for different values of interface-averaged perturbation as shown in Fig. S3 for the case of the real part of $d_{||}$ component for gold-glass interface. Therefore, if we calculate changes in scattering using a small perturbation, quantified with interface-averaged value $\langle \Delta d_i(\mathbf{r}_s)\rangle$ (in our case we choose 1 pm), we can determine the coefficient of proportionality X_i across the spectrum

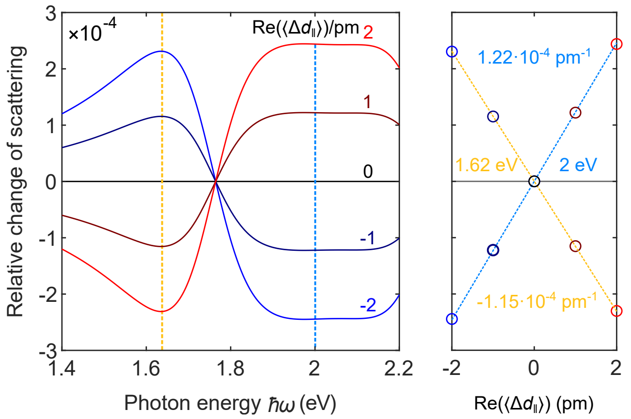

FIG. S3: **Linear scaling of induced change in scattering.** Relative change of scattering is calculated for different interface-averaged values of the $d_{||}$ component perturbation. Induced change scales linearly with the perturbation strength.

$$X_i(\omega) \approx \frac{\Delta_i S(\omega)}{\langle \Delta d_i(\mathbf{r}_s)\rangle}. \tag{S26}$$

Finally, a total relative change of scattering for arbitrary perturbations obtained from the applied voltage V and the perturbation coefficients $\langle \partial_V d_i(\mathbf{r}_s, \omega)\rangle$, is calculated using the following expression

$$\frac{\Delta S(\omega, V)}{S_0} = \sum_i \frac{1}{S_0} \frac{\Delta_i S(\omega)}{\langle \Delta d_i(\mathbf{r}_s)\rangle} \langle \partial_V d_i(\mathbf{r}_s, \omega)\rangle \, V. \tag{S27}$$

In figure S4a we show how the total relative change of scattering, induced by spatially and spectrally dependent d-parameter perturbations, is calculated for the exemplary case of a 140 nm long resonator. In the first column we show the relative change in scattering per 1 pm of interface-averaged perturbation – we refere to these spectra as the "basis functions". In the second column we show spectrally-dependent interface-averaged perturbations for +10 V of applied bias voltage. In the third column we show spectral contributions to the relative change of scattering induced by spatially and spectrally dependent d-parameter perturbations obtained by multiplying the corresponding curves in the first two columns. Additionally, each of the contributions in the first column can be expressed in terms of the influence on the scattering amplitude and eigenfrequency of the system as stated with equation (2) in the main text. First, assuming a lossy harmonic oscillator as a model for a plasmonic nanoresonator, both unperturbed resonance position ω_{r0} and linewidth γ_{r0} are obtained by fitting the simulated scattering spectrum using a Lorentzian function modified with a linear background as shown in Fig. S4b. Subsequently, each of the basis functions is fitted using the relative change of the Lorentzian

$$\frac{1}{S_0} \frac{\Delta_i S(\omega)}{\langle \Delta d_i(\mathbf{r}_s)\rangle} \approx \frac{1}{\langle \Delta d_i(\mathbf{r}_s)\rangle} \left\{ \frac{\Delta A_i}{A_0} + \frac{L_0}{A_0} \left[2(\omega - \omega_{r0})\Delta\omega_{ri} - \frac{\gamma_{r0}}{2} \Delta\gamma_{ri} \right] \right\}, \tag{S28}$$

with fitting parameters $\frac{\Delta_i A}{A_0}$, $\Delta_i \omega_r, \Delta_i \gamma_r$. Fitting is performed only in a spectral interval up to 2 eV, as the simulated relative change shows influences of an additional mode around 2.3 eV. We show fitting parameters for each of d-parameter components for resonators with varying lengths from 80 nm to 200 nm in Fig. S5. From obtained fitting parameters across the spectrum, we can retrieve values for the experimentally realized resonators, and construct the corresponding basis functions.

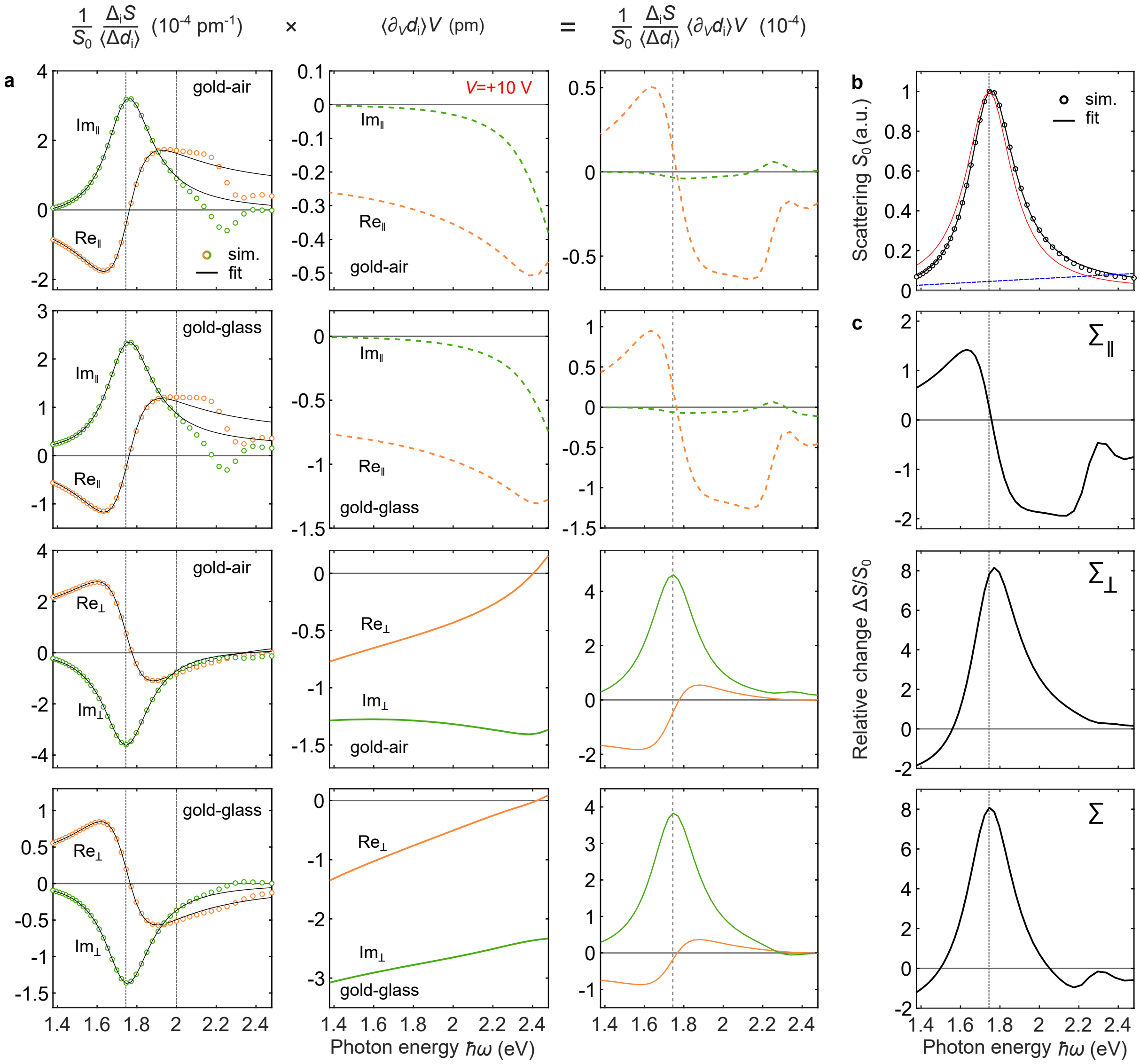

FIG. S4: **Influence of the d-parameter perturbations on the plasmonic resonance.** **a**, Schematics for calculating the total induced relative change of scattering for 140 nm long resonator. In the first column we plot the relative changes of scattering per 1 pm of interface-averaged perturbation for a real (orange) and an imaginary (green) d-parameter perturbation, at a gold-air and a gold-glass interface for an in-plane (dashed) and an out-of-plane (solid) component. For the interfaces as presented in the first column, in the second column we plot corresponding voltage-induced interface-averaged perturbations when bias voltage of +10V is applied. In the third column we show spectral contributions to the relative change of scattering induced by spatially and spectrally dependent d-parameter perturbations obtained by multiplying corresponding curves in the first two columns. **b**, Scattering resonance (black circles) is fitted using Lorentzian (red) modified with linear background (blue), which yield a total curve (black). **c**, Total relative change of scattering produced by summing: all in-plane component terms (up), all out-plane component terms (middle), all of the terms (down). Relative changes of scattering in the first column of **a** are fitted using the relative change of Lorentzian, with fitting parameters $\frac{\Delta_i A}{A_0}$, $\Delta_i \omega_r, \Delta_i \gamma_r$.

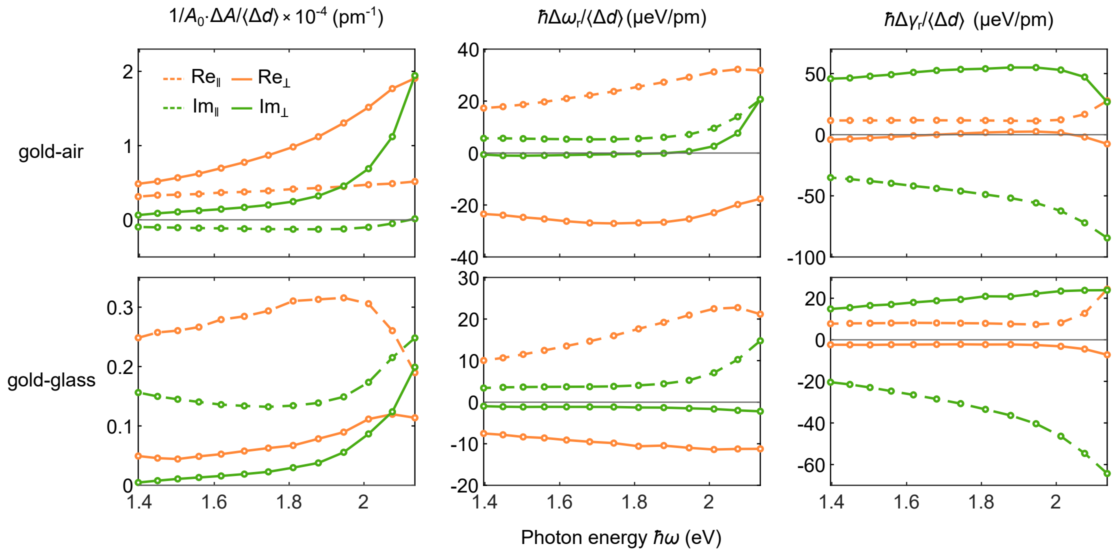

FIG. S5: **Resonance perturbations induced by d-parameters.** Characteristic quantities describing the perturbation of Lorentzian resonance $\frac{\Delta_i A}{A_0}$(left), $\Delta_i\omega_r$ (middle) and $\Delta_i\gamma_r$ (right) per 1 pm of d-parameter perturbations $\langle\Delta d_i\rangle$ for gold-air interface (up), and gold-glass interface (down).

1.10 Summary and workflow of calculation method

The aim of the computation method is to determine the total spectral change in scattering resulting from the application of a voltage $\Delta S(\omega, V)$, with a microscopic origin in locally perturbed d-parameters $\Delta d_i(\omega, \mathbf{r}_s, V)$. The workflow diagram depicted in Fig. S6 starts with the definition of geometry and choice of materials within the FEM model. The gold permittivity ε_m is fitted using the Drude model permittivity ε_D, subsequently subtracted from the total permittivity to derive the background ε_{bm}, associated with interband contributions. This fitting process yields the plasma frequency ω_{p0} and bulk electron density n_0. Subsequently, electrostatic simulations are conducted. The resulting induced surface electron density $\Delta\eta_0(\mathbf{r}_s, V)$ at a surface point $\mathbf{r}_s$ exhibits a linear dependency with respect to the applied voltage V. Consequently, the shape of the induced surface charge or electron distribution remains constant, expressible through the surface capacitance $C_s(\mathbf{r}_s)$. We compute $\Delta\eta_0(\mathbf{r}_s, V)$ for voltages similar to those applied in experimental conditions ($V \approx \pm 10$ V).

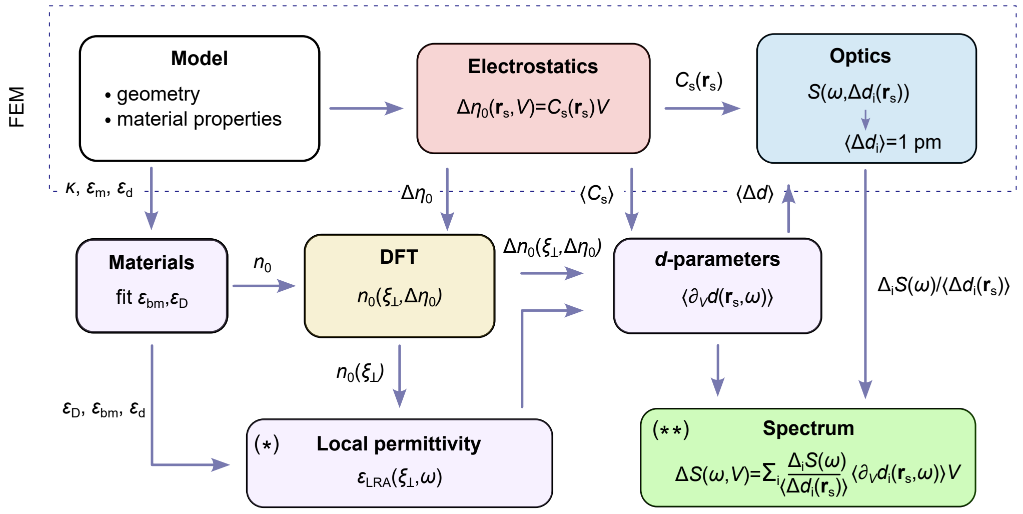

FIG. S6: **Workflow diagram of calculation method.** Two assumptions are denoted with (*) and (**).

Following this, we conduct Density Functional Theory (DFT) calculations for an 8 nm thin slab of jellium, incorporating n_0 as one of the inputs. To perturb the system, we add $q_e\Delta\eta_0$, with a magnitude similar to that

obtained from the electrostatic simulations. Subsequently, we calculate electron densities $n_0(\xi_\perp, \Delta\eta_0)$. The spatially-dependent induced electron density $\Delta n_0(\xi_\perp, \Delta\eta_0)$ maintains a fixed shape and scales linearly with $\Delta\eta_0$. We verify this by examining $\Delta n_0(\xi_\perp, \Delta\eta_0)$ for various values of $\Delta\eta_0$. As a result, we express the spatial dependence of $\Delta n_0(\xi_\perp)$ using the function $p(\xi_\perp)$.

We utilize DFT data to compute the spatially-dependent local permittivity $\varepsilon_{\mathrm{LRA}}(\xi_\perp)$ for gold-air and gold-glass interfaces. This calculation is based on the assumption denoted with (*): *"Background permittivity $\varepsilon_{\mathrm{b}}(\xi_\perp)$ follows the shape of $n_0(\xi_\perp)$, transitioning smoothly between $\varepsilon_{\mathrm{bm}}$ and the surrounding permittivity ε_{d}."*. Subsequently, we determine interface-averaged spectrally-dependent d-parameters perturbation coefficients $\langle \partial_V d(\mathbf{r}_\mathrm{s}, \omega) \rangle$ (as detailed in equations (8) and (10) in the main text) from the local permittivity and the shape of the induced electron density, represented by the function $p(\xi_\perp)$.

Finally, we can calculate the change in scattering due to an arbitrary combination of the d-parameter perturbations using the assumption denoted with (**): *"For every spectral point ω, change in scattering $\Delta_\mathrm{i} S(\omega)$, scales linearly with the introduced perturbation $\Delta d_\mathrm{i}(\mathbf{r}_\mathrm{s})$."* Following this assumption we can introduce the local perturbation in optical simulations simply using $C_\mathrm{s}(\mathbf{r}_\mathrm{s})$. Averaged across the interface perturbation is of the same order of magnitude as obtained from the perturbation coefficients for voltages as applied in the experiment ($\langle \Delta d(\mathbf{r}_\mathrm{s}) \rangle \approx 1\,\mathrm{pm}$). We have checked the validity of this assumption by perturbing the system with different small values of interface averaged perturbation and checking that the induced change scales linearly (see Fig. S4). We introduce the same spatially dependent perturbation for all simulations across the spectrum. Therefore, multiplying the obtained changes in the spectrum $\Delta_\mathrm{i} S(\omega) / \langle \Delta d_\mathrm{i}(\mathbf{r}_\mathrm{s}) \rangle$, by interface-averaged spectrally-dependent d-parameters perturbation coefficients scaled for a voltage of interest $\langle \partial_V d_\mathrm{i}(\omega, \mathbf{r}_\mathrm{s} \rangle V$, we obtain the change in scattering induced by spectrally and spatially dependent perturbation i. Summing up all contributions we obtain the total spectral change in scattering.

1.11 Phenomenological nonlocal response

To include a nonlocal response into our analysis we follow approaches outlined in Refs. [56, 57]. In general, polarization at an arbitrary point $\mathbf{r}$ is described by the following expression:

$$\mathbf{P}(\mathbf{r}) = \varepsilon_0 \int \chi(\mathbf{r}, \mathbf{r}') \mathbf{E}(\mathbf{r}') \mathrm{d}\mathbf{r}', \tag{S29}$$

where $\chi(\mathbf{r}, \mathbf{r}')$ is nonlocal susceptibility tensor. Following the phenomenological approach by Ginzburg and Zayats [57], we take the nonlocal susceptibility tensor in a scalar form as a product

$$\chi(\mathbf{r}, \mathbf{r}') = \chi_{\mathrm{LRA}}(\mathbf{r}') f(\mathbf{r}, \mathbf{r}'), \tag{S30}$$

of $\chi_{\mathrm{LRA}}(\mathbf{r}')$, a local susceptibility, and $f(\mathbf{r}, \mathbf{r}')$, a Gaussian scalar function given by the following expression:

$$f(\mathbf{r}, \mathbf{r}') = A e^{-\frac{(\mathbf{r}-\mathbf{r}')^2}{2\xi_{\mathrm{NL}}^2}}. \tag{S31}$$

Here, A is a normalization factor. The function f carries the response information from the surrounding environment. Its width, represented by ξ_{NL}, defines the effective range of nonlocality, comparable to, for instance, the spread of a delocalized electron wave function. As demonstrated in Ref. [56], by comparing nonlocality in the homogeneous electron gas to the hydrodynamic model, one finds $\xi_{\mathrm{NL}} \propto v_\mathrm{F}/\omega$, where v_F represents the Fermi velocity. Typically, it takes values of approximately 3 Å at 2 eV for a free electron gas characterized with a density $n_0 = 5 \cdot 10^{28}\,\mathrm{m}^{-3}$.

To see the impact of nonlocal contributions we can Taylor expand the electric field $\mathbf{E}(\mathbf{r}')$ in the vicinity of the point of interest $\mathbf{r}$ as

$$E_\mathrm{i}(\mathbf{r}') = E_\mathrm{i}(\mathbf{r}) + \sum_{j_1} \partial^{(1)}_{\mathrm{j}_1} E_\mathrm{i}(\mathbf{r}) \Delta r_{\mathrm{j}_1} + \frac{1}{2} \sum_{j_1, j_2} \partial^{(2)}_{\mathrm{j}_1, \mathrm{j}_2} E_\mathrm{i}(\mathbf{r}) \Delta r_{\mathrm{j}_1} \Delta r_{\mathrm{j}_2} + ... \tag{S32}$$

where $\Delta\mathbf{r} = \mathbf{r}' - \mathbf{r}$. To the second order, the polarization can be expressed as

$$P_\mathrm{i}(\mathbf{r}) \approx \varepsilon_0 \Big[E_\mathrm{i}(\mathbf{r}) \int \chi_\mathrm{LRA}(\mathbf{r}') f(\mathbf{r},\mathbf{r}') \mathrm{d}\mathbf{r}' + \sum_{j_1} \partial^{(1)}_{\mathrm{j}_1} E_\mathrm{i}(\mathbf{r}) \int \chi_\mathrm{LRA}(\mathbf{r}') f(\mathbf{r},\mathbf{r}') \Delta r_{\mathrm{j}_1} \mathrm{d}\mathbf{r}' + \frac{1}{2} \sum_{j_1,j_2} \partial^{(2)}_{\mathrm{j}_1,\mathrm{j}_2} E_\mathrm{i}(\mathbf{r}) \int \chi_\mathrm{LRA}(\mathbf{r}') f(\mathbf{r},\mathbf{r}') \Delta r_{\mathrm{j}_1} \Delta r_{\mathrm{j}_2} \mathrm{d}\mathbf{r}' + ... \Big], \tag{S33}$$

In the bulk, the local response is independent of the position ($\chi_\mathrm{LRA}(\mathbf{r}') = \chi_\mathrm{LRA}$) and can be taken outside of the integral. Furthermore, from the symmetry of the function f the first-order terms and the second-order cross terms vanish and the susceptibility reduces to

$$\mathbf{P}(\mathbf{r}) \approx \varepsilon_0 \chi_\mathrm{LRA} \mathbf{E}(\mathbf{r}) + \frac{1}{2} \varepsilon_0 \chi_\mathrm{LRA} \xi^2_\mathrm{NL} \nabla^2 \mathbf{E}(\mathbf{r}) + ... \tag{S34}$$

Without a strong variation of the electric field across the distance ξ_NL, the response is simply a local response. Due to the symmetry of the function f all odd order momenta are zero.

What has been considered so far is nonlocality due to spatial variation of the electric field in the vicinity of the point of interest. However, it is also important to consider the case when the material properties change rapidly as it is the case of material interfaces, where even if the field is slowly varying we will obtain a nonlocal influence from the first term of Eq. (S33). Therefore, the modified local-permittivity is a convolution of the initial local permittivity and the nonlocal function, given by the following expression:

$$\varepsilon^\mathrm{NL}_\mathrm{LRA}(\xi_\perp) = \int \varepsilon_\mathrm{LRA}(\xi'_\perp) f(\xi_\perp, \xi'_\perp) \mathrm{d}\xi'_\perp. \tag{S35}$$

Therefore, the nonlocal form of the $d_\perp$ component perturbation coefficient is

$$\frac{\partial d_\perp(\mathbf{r}_\mathrm{s}, V)}{\partial V} \approx \frac{\varepsilon_\mathrm{d}\varepsilon_\mathrm{m}}{\varepsilon_\mathrm{d} - \varepsilon_\mathrm{m}} \frac{\omega_\mathrm{p}^2}{\omega^2 + i\gamma\omega} \frac{C_\mathrm{s}(\mathbf{r}_\mathrm{s})}{q_\mathrm{e}} \frac{1}{n_0} \int_{-\infty}^{\infty} d\xi_\perp \frac{p^\mathrm{NL}(\xi_\perp)}{\varepsilon^\mathrm{NL}_\mathrm{LRA}(\xi_\perp)^2}, \tag{S36}$$

where $p^\mathrm{NL}(\xi_\perp)$ is given by the following expression

$$p^\mathrm{NL}(\xi_\perp) = \int p(\xi'_\perp) f(\xi_\perp, \xi'_\perp) \mathrm{d}\xi'_\perp. \tag{S37}$$

Interestingly, higher order nonlocal contributions also come from even terms unlike in the bulk. This is due to the asymmetry of the local response function and, thus, the first moment being nonzero, see Eq. (S33).

We can now test how this type of nonlocality modifies the local permittivity, and subsequently $\partial_V d_\perp$. In Fig. S7a we plot the local permittivity calculated from the equilibrium electron density with and without modification by phenomenological nonlocal response, taking $\xi_\mathrm{NL} = 1.1$ Å. We can see that the local permittivity is smeared out by nonlocality and is most strongly impacted in the region where the local permittivity changes significantly. Already few Angstrom into the metal or into the dielectric, the modified permittivity approaches values of the local permittivity. Using the modified local permittivity we calculate $\partial_V d_\perp$ for a gold-glass interface as shown in Fig. S7b. By gradually increasing the width of the nonlocal function from 0 up to 3.1 Å, we modify more strongly the local permittivity and as a result we change the perturbation coefficients. Namely, the imaginary part diminishes, while the real part increases in magnitude and becomes flat over the spectrum. As a result the relative change in scattering due to out-of-plane perturbation is significantly impacted, as shown in Fig. S7c, leading to a less pronounced change in loss and stronger blueshift for positively charged resonator.

In conclusion, already this basic, phenomenological consideration of nonlocality significantly improves the expected outcome. Nevertheless, to obtain a full picture of true microscopic dynamics, one has to determine the exact nonlocal susceptibility tensor.

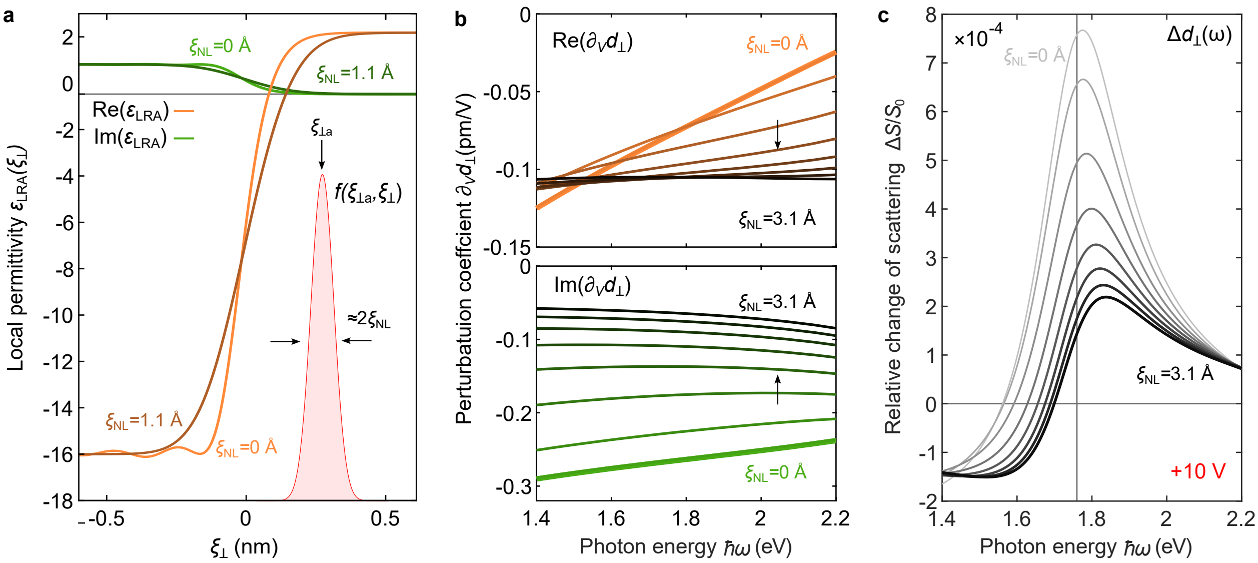

FIG. S7: **Modified out-of-plane response due to nonlocality.** **a**, Spatial dependency of local permittivity on gold-glass interface modified by nonlocal function f characterized with width ξ_{NL} of 0 Å (orange and green curves, initial local permittivity) and 1.1 Å (dark orange and dark green curves, modified local permittivity). **b**, Real (up) and imaginary (down) part of spectrally-dependent interface-averaged perturbation coefficient of the $d_\perp$ component for different widths of nonlocal function ranging from 0 Å (light curves) up to 3.1 Å (dark curves). **c**, Resulting relative change of scattering due to perturbation of out-of-plane response for different widths of nonlocal function ranging from 0 Å(light gray curve) up to 3.1 Å(dark gray curve).

1.12 Dimer antenna by Li *et al.* [29]

We use the framework of surface-response functions in combination with our LRA spill-out model, to analyze the voltage induced response from silver dimer antenna as sketched in Fig. S8a, exhibiting a narrow gap and a sharp tip. This system, originally introduced by Li and co-workers in Ref. [29], promises large scattering modulation for relatively small bias voltages. Unlike in the original study, we apply a bias voltage V to the part of the antenna with the sharp tip. We employ the electrostatic FEM solver and for $V = +1\,\mathrm{V}$ we obtain the induced surface electron density $\Delta\eta_0(\mathbf{r}_\mathrm{s})$ as shown in Fig. S8b. A total charge of $-26\,\mathrm{e}^-$ is retrieved by integrating $\Delta\eta_0(\mathbf{r}_\mathrm{s})$ over the surface of biased antenna arm, with $-8\,\mathrm{e}^-$ on the Ag-BN interface in the gap and only $-0.5\,\mathrm{e}^-$ on the tip apex. To inspect the optical response, the antenna is excited with a plane wave of amplitude E_0, polarized along the long axis of the antenna. As shown in Fig. S8c, the antenna exhibits two modes (at 1.33 eV, and 1.53 eV) localized in the gap, with strong field enhancements $|\mathbf{E}|\,/|\mathbf{E}_0|$ at the apex of the tip ($|\mathbf{E}|\,/|\mathbf{E}_0| \approx 1800$ at 1.33 eV, and $|\mathbf{E}|\,/|\mathbf{E}_0| \approx 1300$ at 1.53 eV). In Fig. S8d we plot the scattering spectrum, featuring two peaks at the modal frequencies. To inspect the impact of charging on these resonances we perturb the system with spatially varying d-parameters $\Delta d_\mathrm{i}(\mathbf{r}_\mathrm{s})$, characterized with 1 pm of interface-averaged value at the biased antenna arm ($\langle\Delta d_\mathrm{i}(\mathbf{r}_\mathrm{s})\rangle = 1\,\mathrm{pm}$) and calculate the relative change of scattering. As shown in Fig. S8e, this is done for the real and the imaginary parts of the parallel and the perpendicular components, for both Ag-vacuum interface (top), and Ag-BN interface (bottom). Note that since this is a coupled system, a small spectral change in one of the resonances will impact the other resonance, and we can no longer, as in the case of the single resonator, make a simple analysis based on the spectral shape of the relative change. Nevertheless, from the retrieved curves we can roughly state a trend. Similar to the single resonator, we can see that the imaginary parts mostly influence resonance widths, with maximum changes on resonance frequencies, while the real parts mostly impact the spectral positions. This takes place for both perturbations at both interfaces, although Ag-BN induced changes are roughly 100 times larger than Ag-vacuum, implying a maximal change of $\approx 50\%$. In the case of Ag-vacuum interface, for the same surface-averaged perturbation, induced change is $\approx 0.3\%$. To calculate the total change, we employ our method outlined in Supplementary Section 1.10. Alongside spectral changes per 1 pm of perturbation $\Delta_\mathrm{i}S(\omega)/\langle\Delta d_\mathrm{i}(\mathbf{r}_\mathrm{s})\rangle$, we calculate corresponding perturbation coefficients $\langle\partial_V d_\mathrm{i}(\mathbf{r}_\mathrm{s},\omega)\rangle$ using Eq. (S36) and Eq. (8) from the main text (see Fig. S8f). Multiplying the relative

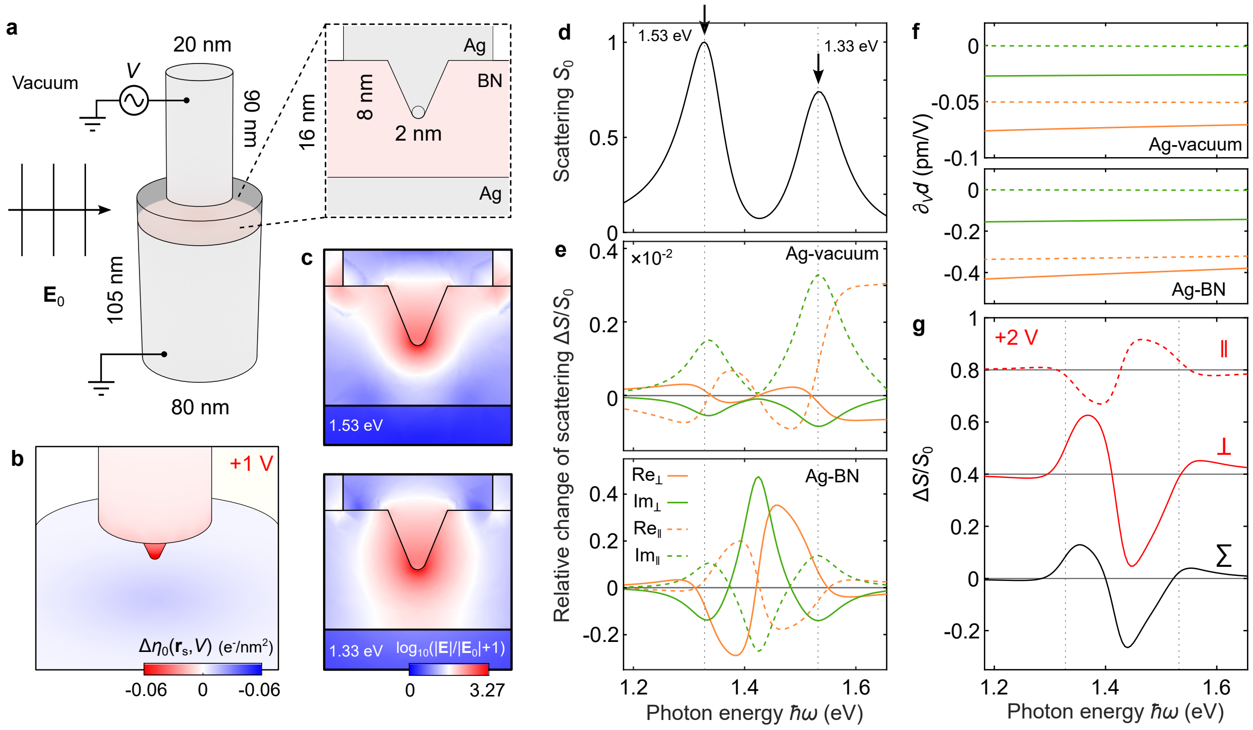

FIG. S8: **Asymmetric dimer antenna. a**, Sketch and dimensions of the silver dimer antenna with narrow gap and sharp tip. **b**, Induced surface electron density for +1 V of applied bias voltage. **c**, Field enhancement plots for two modes at 1.53 eV (up) and 1.33 eV (down). **d**, Scattering spectrum of the unperturbed antenna. **e**, Simulated relative change of scattering $\Delta S/S_0$ for 1 pm of introduced surface-averaged d-parameter perturbations. **e**, Simulated relative change of scattering $\Delta S/S_0$ for 1 pm of introduced surface-averaged d-parameter perturbations. **f**, Spectral dependency of surface-averaged d-parameter perturbation coefficients for the silver-vacuum and silver-BN interface, calculated with (S1) and eq. (8) from the main text. The $d_\perp$ component contributions are depicted with a solid lines, while the $d_\parallel$ components are represented with dashed lines. Real parts are given in orange, and imaginary parts in green. **g**, Calculated total relative change of scattering for +2 V of applied bias voltage by considering: only the in-plane component contributions (top), only the out-of-plane component contributions (middle), all contributions (bottom).

change of scattering per unit perturbation, with corresponding perturbation coefficient scaled for a voltage of interest, we can retrieve the change induced by spatially and spectrally dependent perturbation. Summing terms corresponding to the parallel (perpendicular) component, we obtain the dashed (solid) red curve in Fig. S8g. We can see that the in-plane and out-of-plane perturbations introduce contrasting expectations. Summing the contributions from the two sources, we obtain the total change represented with the black curve in panel d, resembling the out-of-plane response which dominates over the in-plane response. Similar result for the total change is retrieved in the original paper where authors employ quantum-hydrodynamic modeling.

2 Experiment

2.1 Measurement principle

The measurement principle is outlined in Fig. S9. Assuming that we have a Lorentzian resonance as shown in panel a, for which upon applying a bias voltage V resonance position ω_{r0} changes such that, a positive/negative bias leads to a resonance redshift/blueshift by a small amount ($\Delta\omega_r = aV$), then the expected change of scattered power ΔP will have a spectral shape as shown in panel b. The biggest change will take place on the slopes of the resonance, with no change occurring at the position of the resonance. For a positive bias (red curves in panel b) the change will be positive on the red side, and negative on the blue side of the resonance frequency, with the opposite being the case for a negative bias (blue curves in panel b). Furthermore, this change will scale linearly with the change in resonance as discussed in SM section 1.4 (see Eq. (S11)), i.e., with applied bias voltage. Therefore, by monitoring ΔP in the vicinity of the resonance we can determine the shape of a change of resonance and from there a change in the resonance position. As the expected changes are relatively small, to detect them we can use a sinusoidal voltage signal $V(t)$ (characterized with amplitude V and frequency ν_0 as shown in panel c) to drive the system and employ a lock-in amplifier. From the linearity of the induced change in resonance frequency, the induced time-varying power signal $\Delta P(t)$ will also have a sinusoidal time-dependence, characterized with an amplitude ΔP and a phase ϕ. In panel *b*, we denote the expected spectral change at different times marked in panel *c*. If we, for example, monitor time-dependent signals on the red/blue side of the resonance at frequencies ω_1/ω_2, equidistant from the resonance frequency, signals at time t_1 will have the same amplitude but different phases as sketched in panel *c*. Namely, for a signal on the red side, there is no phase shift relative to the driving signal, while the signal on the blue side will be phase-shifted by π.

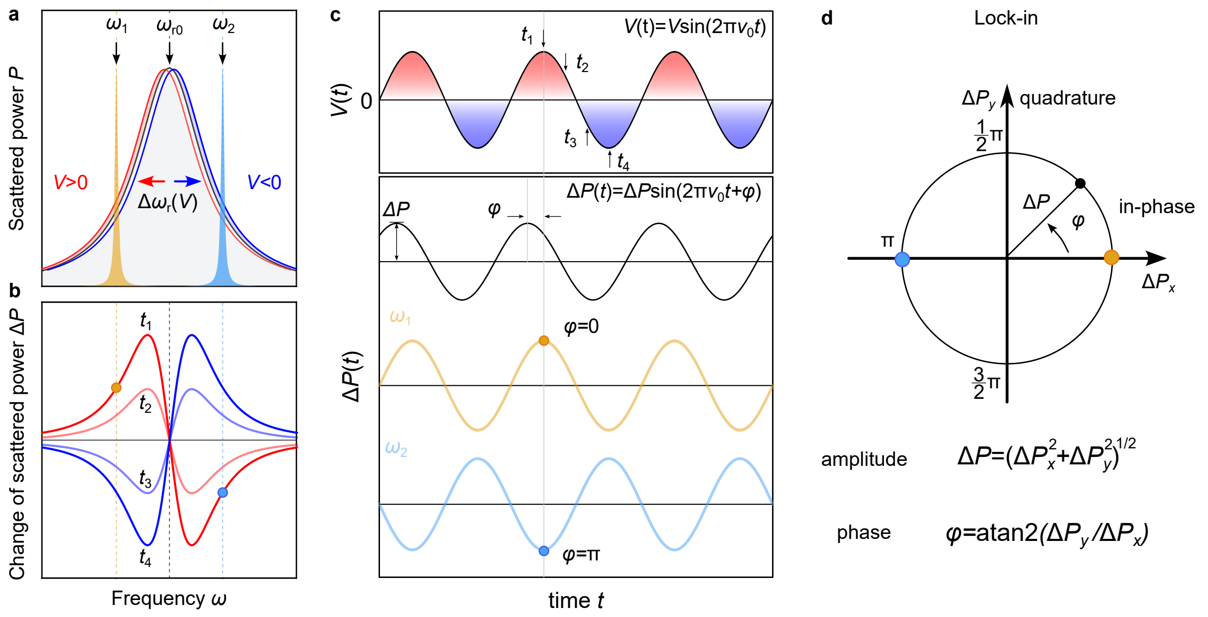

FIG. S9: **Measurement principle.** **a**, A Lorentzian resonance with peak position ω_{r0} is perturbed by a bias voltage V and probed by a spectrally narrow source at optical frequencies $\omega_{1,2}$. **b**, Spectrally dependent change of scattered power for resonance frequency perturbations in panel a. **c**, Time-dependent signals of applied voltage $V(t)$ (top), induced change in scattered power $\Delta P(t)$ (bottom). The black curve corresponds to a general case with amplitude ΔP and phase ϕ. For a signal monitored at optical frequencies $\omega_{1,2}$ as sketched in panels a and b. For a frequency ω_1 (ω_2) represented by the yellow (blue) curve, the signal is in-phase $\phi = 0$ (out-of-phase $\phi = \pi$). **d**, Sketch of a lock-in signals in polar coordinate system.

To get the amplitude ΔP and phase of a signal, one can utilize a phase-sensitive lock-in amplifier. The lock-in gathers an in-phase signal ΔP_x (abscissa axis in panel d) and a quadrature signal ΔP_y (ordinate axis in panel d), enabling determination of ΔP and ϕ as described in panel d. Since our signals should exhibit only 0 or π phase shifts depending on the resonance side we monitor, we expect to observe a purely in-phase signal

across the spectrum. This should provide the shape of induced change as if solely a positive bias voltage were applied, akin to capturing the change snapshot at time t_1. To capture the signal for a negative voltage, equivalent to capturing the change snapshot at time t_4, we can simply multiply the obtained curve by -1.

Nonlinear response

In scenarios where the change in resonance, i.e., the change in scattered power, exhibits nonlinearity response upon applying a bias voltage, we expect to detect lock-in signals at higher harmonics of the driving signal $2\nu_0, 3\nu_0, \ldots$. For example, assuming the change in scattered signal scales with higher powers of voltage, we can represent it as:

$$\begin{aligned}\Delta P(t) =& a_1 V \sin(\omega_0 t) + a_2 V^2 \sin^2(\omega_0 t) \\ &+ a_3 V^3 \sin^3(\omega_0 t) + a_4 V^4 \sin^4(\omega_0 t) + \ldots\end{aligned} \tag{S38}$$

Here, $\omega_0 = 2\pi\nu_0$, and by employing trigonometric identities, we can express it in terms of harmonics:

$$\Delta P(t) = A_0 + A_1 \sin(\omega_0 t) + A_2 \cos(2\omega_0 t) + A_3 \sin(3\omega_0 t) + \ldots \tag{S39}$$

Where coefficients $A_{\mathrm{i}}, i = \{0, 1, 2, \ldots\}$ are given by:

$$\begin{aligned}A_0 &= \frac{a_2}{2}V^2 + \frac{3a_4}{8}V^4 + \ldots \\ A_1 &= a_1 V + \frac{3a_3}{4}V^3 + \ldots \\ A_2 &= -\frac{a_2}{2}V^2 - \frac{a_4}{8}V^4 + \ldots \\ A_3 &= -\frac{a_3}{4}V^3 + \ldots \\ A_4 &= \frac{a_4}{8}V^4 + \ldots \\ &\ldots\end{aligned} \tag{S40}$$

Hence, observing the dependence of the lock-in signal at the first harmonic on the applied voltage amplitude provides insight into all odd-order contributions (represented by coefficients $a_1, a_3, \ldots$). Similarly, monitoring the lock-in signal at the second harmonic offers information on all even-order contributions (indicated by coefficients $a_2, a_4, \ldots$).

2.2 Electro-optical setup

To measure the relative change of the scattering, we utilize an electro-optical setup, as depicted in Fig. S10. The setup is divided into two paths using a 50:50 beam splitter, i.e., the excitation path and the detection path. In the excitation path, we employ a tunable laser as the light source operating at an optical frequency ω. The laser light passes through a 300 µm pinhole and is then collimated using a lens positioned before the beam splitter. The iris is positioned before the beam splitter to remove high-angle contributions from the pinhole diffraction. Therefore, a weakly focused beam is used to excite the structure, causing it to scatter light. The light reflected from the sample and scattered by the nanoresonator is guided through the detection beam path. Here, the reflected light is filtered out using a beam block. Additional filtering of the scattered signal is done by refocusing the scattered light to an intermediate image plane and using an iris to remove stray light. The scattered light is then split using a 90:10 beam splitter, dividing the scattered light between the photodetector and the optical powermeter. The powermeter provides a direct readout, allowing to obtain the unperturbed part of the scattered power P_0. The signal from the photodetector is sent to a lock-in amplifier, enabling the accurate measurement of the change in scattered power ΔP.

2.3 Scattering measurements

To determine the scattering cross-section, it is necessary to measure both the power scattered by the structure, P_{s}, and the excitation intensity, $I \approx P_{\mathrm{in}}/r^2\pi$, where P_{in} represents the incoming power and r is the radius of the

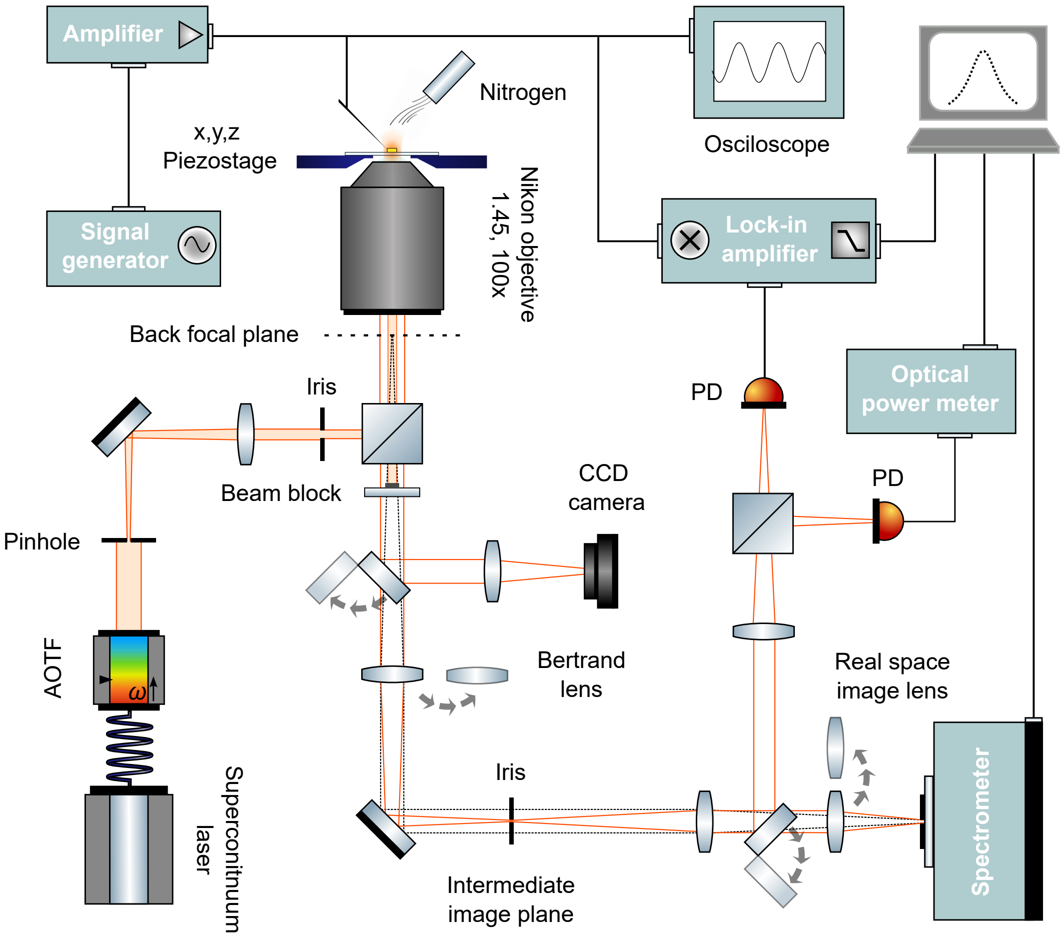

FIG. S10: **Experimental setup.** Schematics of electro-optical setup.

excitation spot. The typical beam size is approximately 1.5 µm, while the length of the longest nanoresonator is 180 nm. Therefore, it is assumed that the intensity is constant across the nanoresonator which justifies the use of a plane wave as an excitation source in simulations.

The nanoresonator is illuminated with light from the glass side, at angles below the critical angle. The power scattered by the nanoresonator P_s is measured by collecting the scattered light using the same objective that was used for excitation. The collection of light occurs at angles above the critical angle but below the maximum collection angle, which is determined by the numerical aperture (NA) of the objective.

To separate the light reflected from the sample from the scattered light, a circular patch is introduced in the detection beam path. This circular patch effectively removes the directly reflected light. However, it is important to note that a small background signal persists due to the presence of stray light and scattering of light on imperfections present on the substrate. Consequently, the total scattered power, denoted as P, can be expressed as the sum of the power originating from the nanoresonator structure P_s, and the background scattered power P_b (see Fig. S11). The background signal is filtered out to a large extent by focusing the light onto an intermediate plane and implementing an iris to block light originating from scattering sources that are not in the proximity of the nanoresonator. To evaluate the impact of the residual background resulting from other scattering sources, we conduct measurements on a flat glass substrate while keeping the beam block in position. By measuring the signal under these conditions, we observe that the background power is negligible compared to the power scattered by the nanoresonator structure – it accounts only for a few percent of the total scattered power as shown in Fig. S11, indicating that $P_s \approx P$.

In order to determine the incoming intensity I, we begin by measuring the power of light that is reflected from a flat glass substrate, denoted as P_r, in the absence of the beam block. Assuming that the reflectance R remains constant across the spectral range, the incoming power can be determined up to a constant factor R as

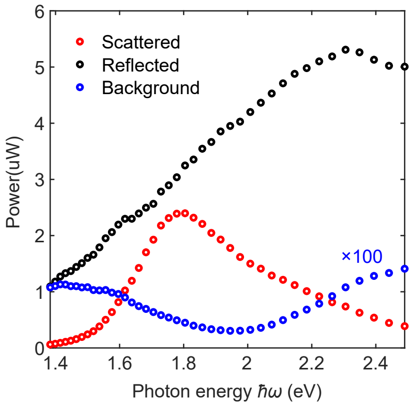

FIG. S11: **Measured scattered power.** Power of scattered signal (red), reflected signal (black) and background signal (blue).

$P_r = RP_{in}$.

2.4 Characteristics of the excitation source

Spatial shape

To characterize the excitation source both spectrally and spatially, we direct the beam reflected from the sample directly to the spectrometer (see S10). Initially, we measure the shape of the beam, as depicted in Fig. S12a. By fitting a Gaussian function to the beam profiles in both the x and y directions, which are passing through the intensity maximum, we extract the corresponding beam widths, r_x and r_y. These beam widths are expressed in pixels, as illustrated in Fig. S12b. To obtain the frequency dependent beam size, denoted as $\bar{r}$, we calculate the average of these two beam widths. Subsequently, we calculate the spectral shape of the average intensity, denoted as I, which is inversely proportional to the square of the beam size. It can be mathematically expressed as $I \approx CP_r/\bar{r}^2$. Therefore, the intensity and scattering cross-section are determined up to a constant factor C.

$$S = C \cdot \sigma_{sca} \approx \bar{r}^2 \frac{P}{P_r}. \tag{S41}$$

We refer to S as scattering signal. As a result, the relative change in the scattering cross-section i.e. scattering signal is equal to the relative change of the scattered power, that is

$$\frac{\Delta\sigma_{sca}}{\sigma_{sca0}} = \frac{\Delta S}{S_0} \approx \frac{\Delta P}{P_0}, \tag{S42}$$

where P_0 denotes the unperturbed scattered power. This provides us with the possibility to relate experimental data directly to the model.

It is important to note that each of the measured quantities is influenced by the setup transfer function T_s. However, when we take a quotient of these quantities, the effects of the transfer function cancel out.

Spectral shape

In an ideal scenario, it would be preferable to employ an excitation source, centered at frequency ω, with a zero spectral line width to precisely investigate the response of the nanoresonator at exactly this optical frequency. However, in our setup, the excitation laser exhibits a non-zero spectral line width, as depicted in Fig. S12c. This introduces spectral distortion in the measurements due to the convolution of the true scattering cross-section and the intensity given by a Gaussian function $I_G(\omega; \omega_l, \sigma) = I(\omega) \cdot G(\omega; \omega_l, \sigma)$, where ω_l represents the peak position and σ represents the width of the Gaussian function.

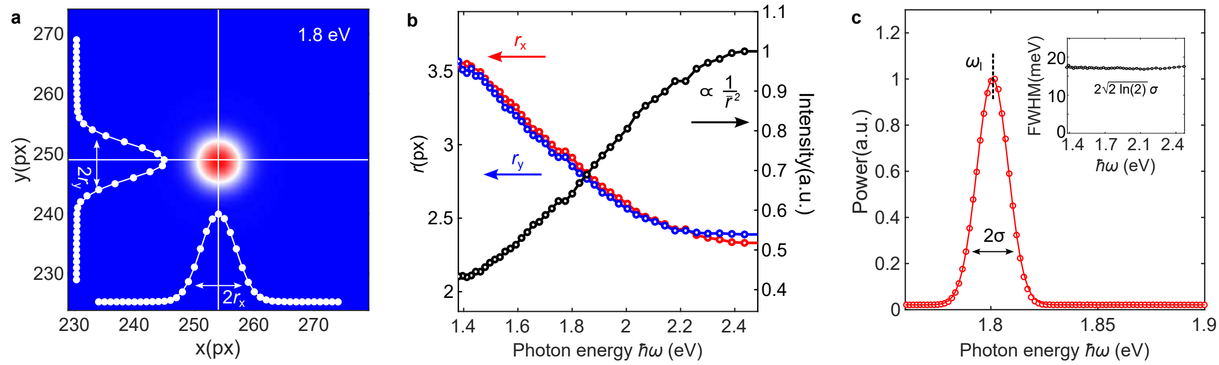

FIG. S12: **Characteristics of the excitation source. a** An image of the excitation beam is captured at 1.8 eV, after it has been reflected from a glass substrate. The image shows the beam profiles in both x and y direction. Gaussian curves are fitted to these profiles to determine the beam parameters. **b** The beam sizes in the x and y directions are measured for different excitation frequencies. The average of the two beam sizes is used to calculate the intensity of the excitation source. **c** The source spectrum obtained for a frequency of 1.8 eV. A Gaussian curve is fitted to the spectrum to determine the spectral line width. The inset shows the spectral line width in dependency of the excitation frequency.

Consequently, the measured power will be affected by this spectral distortion

$$\begin{aligned} P'(\omega) = (\sigma_{\text{sca}} * I_G)(\omega) &= \int_{-\infty}^{\infty} \sigma_{\text{sca}}(\omega') \cdot I(\omega') \cdot G(\omega'; \omega, \sigma) d\omega' \\ &= \int_{-\infty}^{\infty} P(\omega') \cdot G(\omega'; \omega, \sigma) d\omega' = (P * G)(\omega). \end{aligned} \tag{S43}$$

To retrieve the true scattered power $P(\omega)$ we need to deconvolute the measured signal. Figure S13 illustrates the scattering signal taking into account the excitation source characteristics. The plot presents two scenarios: one considering only the beam size (black circles) and another (blue line) considering both the beam size and the spectral line width. The results show that the beam size influences the shape of the calculated spectrum, while the spectral line width of the excitation source has minimal impact.

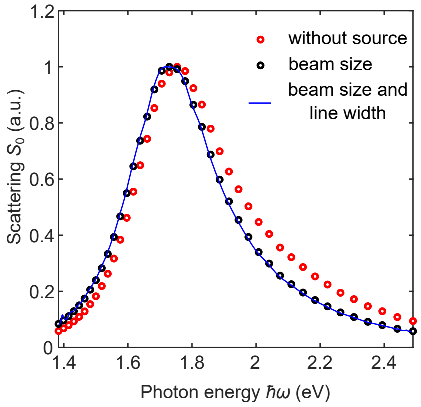

FIG. S13: **Measured scattering spectrum.** The scattering spectrum is plotted with and without considering the influence of the source characteristics. The red circles correspond to the directly measured spectrum. The black circles represent the corrected spectrum, considering the non-constant intensity spectrum due to the dispersive beam size. Additionally, the blue curve represents the spectral distortion caused by the non-zero line width of the excitation source.

2.5 Exemplary lock-in measurements

It is expected that the charge effect observed in lock-in measurements will only manifest at the fundamental frequency ν_0, i.e., the frequency of the driving signal. Additionally, the signal should remain constant across the entire spectrum up to a frequency determined by the RC time of the system. At sufficiently low frequencies, only the in-phase component should be present, exhibiting a linear relationship with the applied voltage. These expectations have been experimentally validated. In Fig. S14a we show the relative change in the scattering spectrum, measured at a driving frequency of $\nu_0 = 24\,\text{kHz}$ with an applied voltage of 10 V, clearly exhibits a purely in-phase component. Panel b) demonstrates the absence of a lock-in signal at the second harmonic frequency $2\nu_0$. Furthermore, we test if the signal changes for a nanoresonator coated with a thin layer of AlOx and show result in panel c. Coating is done using atomic layer deposition (similar to the procedure in Ref. [67]).

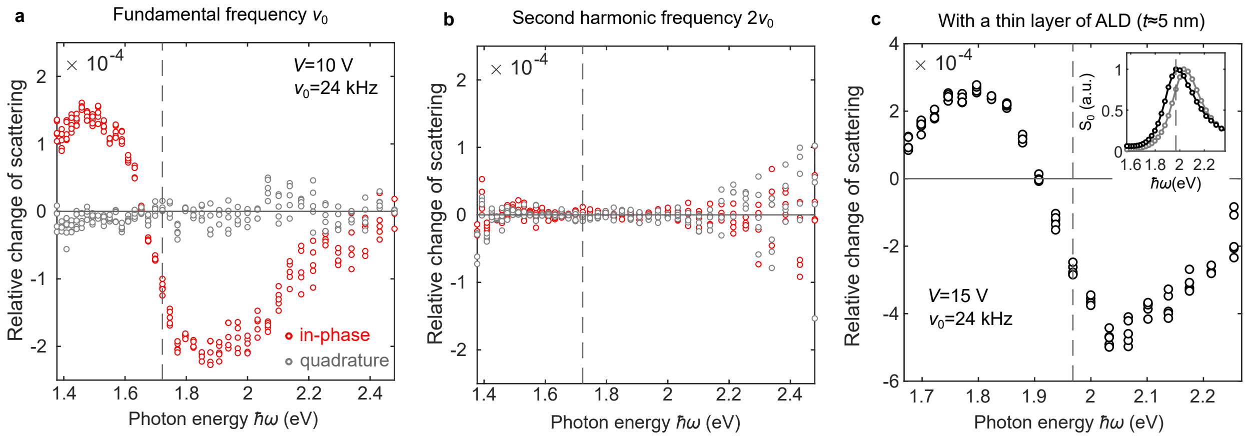

FIG. S14: **Exemplary lock-in measurements.** Spectral dependency of relative change of scattering for both in-phase (red) and quadrature (gray) component for an applied voltage of $V = 10\,\text{V}$ at a driving(fundamental) frequency $\nu_0 = 24\,\text{kHz}$ (panel **a**) and at the second harmonic frequency $\nu_0 = 2 \times 24\,\text{kHz}$ (panel **b**). **c**, Response of a 100 nm long nanoresonator covered with a thin layer of ALD (5 nm). In the inset we show inital spectrum (gray) and spectrum with ALD (black). Measurement was done with 15 V of an applied voltage at 24 kHz frequency.

2.6 Estimating the $\Delta d_\perp$

By employing our analysis tool established in section 1.9, we can estimate the unknown $d_\perp$. As the d-parameter perturbation coefficients are smoothly varying functions across the spectrum we can express them using a polynomial expression:

$$\langle \partial_V d_{\mathrm{i}}(\mathbf{r}_{\mathrm{s}}, \omega) \rangle = \langle C_{\mathrm{s}}(\mathbf{r}_{\mathrm{s}}) \rangle \cdot \left(a_{0\mathrm{i}} + a_{1\mathrm{i}}\omega + a_{2\mathrm{i}}\omega^2 + ... \right). \tag{S44}$$

If the d-parameter perturbations $\langle \partial_V d_{\mathrm{i}}(\mathbf{r}_{\mathrm{s}}, \omega) \rangle\, V$ are constant across the spectrum ($a_{\mathrm{ji}} = 0,\ j \neq 0$), then the resulting change in scattering can also be fitted using the relative change of Lorentzian i.e. the resulting spectrum can be fitted using the basis functions. Thus, we have to solve the following over-determined system of equations

$$\frac{\Delta S(\omega, V)}{S_0} = \sum_i \frac{1}{S_0} \frac{\Delta_{\mathrm{i}} S(\omega)}{\langle \Delta d_{\mathrm{i}} \rangle} \langle C_{\mathrm{s}}(\mathbf{r}_{\mathrm{s}}) \rangle\, a_{0\mathrm{i}} V, \tag{S45}$$

where $a_{0\mathrm{i}}$ are unknowns. Note that, we can only perform this procedure and exactly retrieve the unknown d-parameters for either $d_\perp$ or $d_\parallel$ component at one of the interfaces, as the basis functions corresponding to the real/imaginary perturbations at different interfaces are very similar (no independent basis functions in a mathematical sense). Furthermore, in this scenario the resulting parameters that characterize change in a resonance $\Delta A/A_0, \Delta\omega_{\mathrm{r}}, \Delta\gamma_{\mathrm{r}}$ are simply a linear combination of characteristic perturbations $\Delta_{\mathrm{i}} A/A_0, \Delta_{\mathrm{i}}\omega_{\mathrm{r}}, \Delta_{\mathrm{i}}\gamma_{\mathrm{r}}$ per unit perturbation $\langle \Delta d_{\mathrm{i}} \rangle$, scaled for a perturbation of interest $\langle \partial_V d_{\mathrm{i}}(\mathbf{r}_{\mathrm{s}}, \omega) \rangle\, V$. If this is not the case ($a_{\mathrm{ji}} \neq 0$, as it generally is not) the resulting characteristic perturbations get mixed. Then we have to default back to fitting

across all of the spectrum but now considering higher order terms of the polynomial

$$\frac{\Delta S(\omega, V)}{S_0} = \sum_i \frac{1}{S_0} \frac{\Delta_i S(\omega)}{\langle \Delta d_i \rangle} \langle C_s(\mathbf{r}_s) \rangle \left(a_{0i} + a_{1i}\omega + a_{2i}\omega^2 + ... \right) V. \tag{S46}$$

We have tested the method for a case of a single d-parameter (taking into account both real and imaginary part). First we have calculate resulting spectrum with known input value, then using the corresponding basis functions and fitting procedure we have retrieved the input value.

We now apply this method to experimental data. First, we assume that the difference between experimental data and calculated in-plane component can be assigned to an out-of-plane response. The idea behind this is that the $d_{\|}$ component calculated using the LRA is a certain and therefore fix parameter. Other contribution such as conduction through the surface states should exhibit similar spectral shape and therefore cannot explain the resonance broadening. Since we now have four spectrally dependent unknown parameters (real an imaginary parts for two interfaces) we would expect that the resulting data can be fitted using a combination of all of them. Nevertheless, as pointed out earlier, due to the similarity of basis functions in the measured frequency range, we cannot unambiguously retrieve multiple unknown d-parameters. Therefore, we can only estimate the maximum value of the parameters at one of the interfaces by completely neglecting contributions from the other interface.

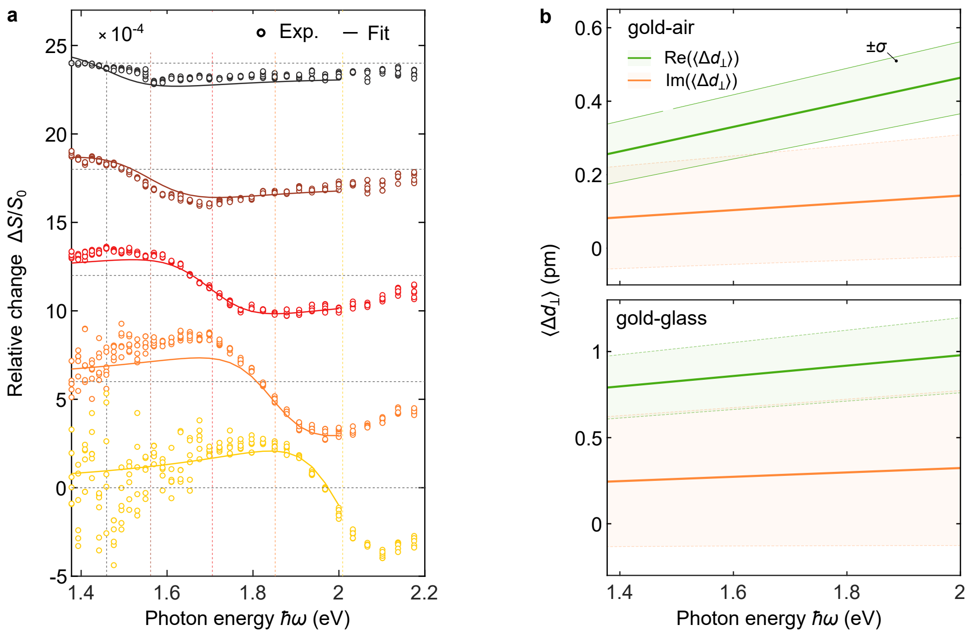

FIG. S15: **Estimating the** $d_\perp$. **a**, total spectra (circles) and resulting curve obtain by fitting the $d_\perp$ at the gold-air interface. **b**, $d_\perp$ parameter obtained by fitting procedure for gold-air interface (up) and gold-glass interface (down).

The results of this fitting estimation is plotted in Fig. S15: panel a shows the fits using only the basis function retrieved for $d_\perp$ of the glass-gold interface. Panel b shows the fitted size of the d-parameter perturbations, all lying between $10^{-1} - 1$ pm, which from absolute size is comparable to calculated d-parameter perturbations.

REFERENCES AND NOTES

1. R. H. Ritchie, Plasma losses by fast electrons in thin films. *Phys. Rev.* **106**, 874–881 (1957).

2. P. Muhlschlegel, H.-J. Eisler, O. J. F. Martin, B. Hecht, D. W. Pohl, Resonant optical antennas. *Science* **308**, 1607–1609 (2005).

3. L. Novotny, Effective wavelength scaling for optical antennas. *Phys. Rev. Lett.* **98**, 266802 (2007).

4. A. G. Curto, G. Volpe, T. H. Taminiau, M. P. Kreuzer, R. Quidant, N. F. van Hulst, Unidirectional emission of a quantum dot coupled to a nanoantenna. *Science* **329**, 930–933 (2010).

5. M. W. Knight, H. Sobhani, P. Nordlander, N. J. Halas, Photodetection with active optical antennas. *Science* **332**, 702–704 (2011).

6. R. Zhang, Y. Zhang, Z. C. Dong, S. Jiang, C. Zhang, L. G. Chen, L. Zhang, Y. Liao, J. Aizpurua, Y. Luo, J. L. Yang, J. G. Hou, Chemical mapping of a single molecule by plasmon-enhanced Raman scattering. *Nature* **498**, 82–86 (2013).

7. J. Kern, R. Kullock, J. Prangsma, M. Emmerling, M. Kamp, B. Hecht, Electrically driven optical antennas. *Nat. Photonics* **9**, 582–586 (2015).

8. M. Celebrano, X. Wu, M. Baselli, S. Großmann, P. Biagioni, A. Locatelli, C. De Angelis, G. Cerullo, R. Osellame, B. Hecht, Mode matching in multiresonant plasmonic nanoantennas for enhanced second harmonic generation. *Nat. Nanotechnol.* **10**, 412–417 (2015).

9. R. Chikkaraddy, B. de Nijs, F. Benz, S. J. Barrow, O. A. Scherman, E. Rosta, A. Demetriadou, P. Fox, O. Hess, J. J. Baumberg, Single-molecule strong coupling at room temperature in plasmonic nanocavities. *Nature* **535**, 127–130 (2016).

10. N. Jiang, X. Zhuo, J. Wang, Active plasmonics: Principles, structures, and applications. *Chem. Rev.* **118**, 3054 (2017).

11. F. Wang, Y. Zhang, C. Tian, C. Girit, A. Zettl, M. Crommie, Y. R. Shen, Gate-variable optical transitions in graphene. *Science* **320**, 206–209 (2008).

12. F. Bonaccorso, Z. Sun, T. Hasan, A. C. Ferrari, Graphene photonics and optoelectronics. *Nat. Photonics* **4**, 611–622 (2010).

13. M. Liu, X. Yin, E. Ulin-Avila, B. Geng, T. Zentgraf, L. Ju, F. Wang, X. Zhang, A graphene-based broadband optical modulator. *Nature* **474**, 64–67 (2011).

14. B. McMillan, L. Berlouis, F. Cruickshank, P. Brevet, Reflectance and electrolyte electroreflectance from gold nanorod arrays embedded in a porous alumina matrix. *J. Electroanal. Chem.* **599**, 177–182 (2007).

15. P. Mulvaney, J. Pérez-Juste, M. Giersig, L. M. Liz-Marzán, C. Pecharromán, Drastic surface plasmon mode shifts in gold nanorods due to electron charging. *Plasmonics* **1**, 61–66 (2006).

16. C. Novo, A. M. Funston, A. K. Gooding, P. Mulvaney, Electrochemical charging of single gold nanorods. *J. Am. Chem. Soc.* **131**, 14664–14666 (2009).

17. T. Miyazaki, R. Hasegawa, H. Yamaguchi, H. Oh-oka, H. Nagato, I. Amemiya, S. Uchikoga, Electrical control of plasmon resonance of gold nanoparticles using electrochemical oxidation. *J. Phys. Chem. C* **113**, 8484–8490 (2009).

18. S. Dondapati, M. Ludemann, R. Muller, S. Schwieger, A. Schwemer, B. Handel, D. Kwiatkowski, M. Djiango, E. Runge, T. Klar, Voltage-induced adsorbate damping of single gold nanorod plasmons in aqueous solution. *Nano Lett.* **12**, 1247–1252 (2012).

19. C. P. Byers, B. S. Hoener, W.-S. Chang, M. Yorulmaz, S. Link, C. F. Landes, Single-particle spectroscopy reveals heterogeneity in electrochemical tuning of the localized surface plasmon. *J. Phys. Chem. B.* **118**, 14047–14055 (2014).

20. A. M. Brown, M. T. Sheldon, H. A. Atwater, Electrochemical tuning of the dielectric function of au nanoparticles. *ACS Photonics* **2**, 459–464 (2015).

21. S. S. E. Collins, X. Wei, T. G. McKenzie, A. Funston, P. C. Mulvaney, Single gold nanorod charge modulation in an ion gel device. *Nano Lett.* **16**, 6863–6869 (2016).

22. B. S. Hoener, H. Zhang, T. S. Heiderscheit, S. R. Kirchner, A. S. De Silva Indrasekara, R. Baiyasi, Y. Cai, P. Nordlander, S. Link, C. F. Landes, W.-S. Chang, Spectral response of plasmonic gold nanoparticles to capacitive charging: Morphology effects. *J. Phys. Chem. Lett.* **8**, 2681–2688 (2017).

23. T. Liu, M. Li, Y. Wang, Y. Fang, W. Wang, Electrochemical impedance spectroscopy of single Au nanorods. *Chem. Sci.* **9**, 4424–4429 (2018).

24. R. A. Maniyara, D. Rodrigo, R. Yu, J. Canet-Ferrer, D. S. Ghosh, R. Yongsunthon, D. E. Baker, A. Rezikyan, F. J. García de Abajo, V. Pruneri, Tunable plasmons in ultrathin metal films. *Nat. Photonics* **13**, 328–333 (2019).

25. T. A. Petach, M. Lee, R. C. Davis, A. Mehta, D. Goldhaber-Gordon, Mechanism for the large conductance modulation in electrolyte-gated thin gold films. *Phys. Rev. B* **90**, 081108 (2014).

26. M. T. Sheldon, J. van de Groep, A. M. Brown, A. Polman, H. A. Atwater, Plasmoelectric potentials in metal nanostructures. *Science* **346**, 828–831 (2014).

27. C. F. Bohren, A. J. Hunt, Scattering of electromagnetic waves by a charged sphere. *Canadian J. Phys.* **55**, 1930–1935 (1977).

28. M. Zapata Herrera, J. Aizpurua, A. K. Kazansky, A. G. Borisov, Plasmon response and electron dynamics in charged metallic nanoparticles. *Langmuir* **32**, 2829–2840 (2016).

29. W. Li, Q. Zhou, P. Zhang, X.-W. Chen, Direct electro plasmonic and optic modulation via a nanoscopic electron reservoir. *Phys. Rev. Lett.* **128**, 217401 (2022).

30. P. J. Feibelman, Surface electromagnetic fields. *Prog. Surf. Sci.* **12**, 287–407 (1982).

31. U. Kreibig, L. Genzel, Optical absorption of small metallic particles. *Surf. Sci.* **156**, 678–700 (1985).

32. J. Tiggesbäumker, L. Köller, K.-H. Meiwes-Broer, A. Liebsch, Blue shift of the mie plasma frequency in Ag clusters and particles. *Phys. Rev. A* **48**, R1749–R1752 (1993).

33. E. Cottancin, G. Celep, J. Lermé, M. Pellarin, J. R. Huntzinger, J. L. Vialle, M. Broyer, Optical properties of noble metal clusters as a function of the size: Comparison between experiments and a semi-quantal theory. *Theoret. Chem. Acc.* **116**, 514–523 (2006).

34. J. Zuloaga, E. Prodan, P. Nordlander, Quantum description of the plasmon resonances of a nanoparticle dimer. *Nano Lett.* **9**, 887–891 (2009).

35. K. J. Savage, M. M. Hawkeye, R. Esteban, A. G. Borisov, J. Aizpurua, J. J. Baumberg, Revealing the quantum regime in tunnelling plasmonics. *Nature* **491**, 574–577 (2012).

36. J. A. Scholl, A. L. Koh, J. A. Dionne, Quantum plasmon resonances of individual metallic nanoparticles. *Nature* **483**, 421–427 (2012).

37. C. Ciracì, R. T. Hill, J. J. Mock, Y. Urzhumov, A. I. Fernández-Domìnguez, S. A. Maier, J. B. Pendry, A. Chilkoti, D. R. Smith, Probing the ultimate limits of plasmonic enhancement. *Science* **337**, 1072–1074 (2012).

38. S. Raza, N. Stenger, S. Kadkhodazadeh, S. V. Fischer, N. Kostesha, A.-P. Jauho, A. Burrows, M. Wubs, N. A. Mortensen, Blueshift of the surface plasmon resonance in silver nanoparticles studied with EELS. *Nanophotonics* **2**, 131–138 (2013).

39. W. Zhu, R. Esteban, A. G. Borisov, J. J. Baumberg, P. Nordlander, H. J. Lezec, J. Aizpurua, K. B. Crozier, Quantum mechanical effects in plasmonic structures with subnanometre gaps. *Nat. Commun.* **7**, 11495 (2016).

40. Y. Yang, D. Zhu, W. Yan, A. Agarwal, M. Zheng, J. D. Joannopoulos, P. Lalanne, T. Christensen, K. K. Berggren, M. Soljačić, A general theoretical and experimental framework for nanoscale electromagnetism. *Nature* **576**, 248–252 (2019).

41. S. Boroviks, Z.-H. Lin, V. A. Zenin, M. Ziegler, A. Dellith, P. A. D. Gonçalves, C. Wolff, S. I. Bozhevolnyi, J.-S. Huang, N. A. Mortensen, Extremely confined gap plasmon modes: When nonlocality matters. *Nat. Commun.* **13**, 3105 (2022).

42. N. A. Mortensen, P. A. D. Gonçalves, F. A. Shuklin, J. D. Cox, C. Tserkezis, M. Ichikawa, C. Wolff, Surface-response functions obtained from equilibrium electron-density profiles. *Nanophotonics* **10**, 3647–3657 (2021).

43. S. Raza, S. I. Bozhevolnyi, M. Wubs, N. A. Mortensen, Nonlocal optical response in metallic nanostructures. *J. Phys. Condens. Matter* **27**, 183204 (2015).

44. D. Jin, Q. Hu, D. Neuhauser, F. von Cube, Y. Yang, R. Sachan, T. S. Luk, D. C. Bell, N. X. Fang, Quantum-spillover-enhanced surface-plasmonic absorption at the interface of silver and high-index dielectrics. *Phys. Rev. Lett.* **115**, 193901 (2015).

45. J. B. Khurgin, Ultimate limit of field confinement by surface plasmon polaritons. *Faraday Discuss.* **178**, 109–122 (2015).

46. T. V. Shahbazyan, Landau damping of surface plasmons in metal nanostructures. *Phys. Rev. B* **94**, 235431 (2016).

47. N. A. Mortensen, Mesoscopic electrodynamics at metal surfaces. *Nanophotonics* **10**, 2563–2616 (2021).

48. A. R. Echarri, P. A. D. Gonçalves, C. Tserkezis, F. J. García de Abajo, N. A. Mortensen, J. D. Cox, Optical response of noble metal nanostructures: Quantum surface effects in crystallographic facets. *Optica* **8**, 710–721 (2021).

49. T. Christensen, W. Yan, A.-P. Jauho, M. Soljačić, N. A. Mortensen, Quantum corrections in nanoplasmonics: Shape, scale, and material. *Phys. Rev. Lett.* **118**, 157402 (2017).

50. P. Lalanne, W. Yan, K. Vynck, C. Sauvan, J.-P. Hugonin, Light interaction with photonic and plasmonic resonances. *Laser Photon. Rev.* **12**, 1700113 (2018).

51. J. Yang, H. Giessen, P. Lalanne, Simple analytical expression for the peak-frequency shifts of plasmonic resonances for sensing. *Nano Lett.* **15**, 3439–3444 (2015).

52. E. Krauss, R. Kullock, X. Wu, P. Geisler, N. Lundt, M. Kamp, B. Hecht, Controlled growth of high-aspect-ratio single-crystalline gold platelets. *Cryst. Growth Des.* **18**, 1297–1302 (2018).

53. J. Meier, L. Zurak, A. Locatelli, T. Feichtner, R. Kullock, B. Hecht, Controlling field asymmetry in nanoscale gaps for second harmonic generation. *Adv. Opt. Mater.* **11**, 2300731 (2023).

54. B. Yan, B. Stadtmüller, N. Haag, S. Jakobs, J. Seidel, D. Jungkenn, S. Mathias, M. Cinchetti, M. Aeschlimann, C. Felser, Topological states on the gold surface. *Nat. Commun.* **6**, 10167 (2015).

55. P. Dreher, D. Janoschka, B. Frank, H. Giessen, F.-J. M. zu Heringdorf, Focused surface plasmon polaritons coherently couple to electronic states in above-threshold electron emission. *Commun. Phys.* **6**, 15 (2023).

56. N. A. Mortensen, Nonlocal formalism for nanoplasmonics: Phenomenological and semi-classical considerations. *Photonic. Nanostruct. Fundam. Appl.* **11**, 303–309 (2013).

57. P. Ginzburg, A. V. Zayats, Localized surface plasmon resonances in spatially dispersive nano-objects: Phenomenological treatise. *ACS Nano* **7**, 4334–4342 (2013).

58. Q. Zhou, W. Li, Z. He, P. Zhang, X.-W. Chen, Quantum hydrodynamic model for noble metal nanoplasmonics. *Phys. Rev. B* **107**, 205413 (2023).

59. COMSOL Multiphysics v. 6.0., www.comsol.com. COMSOL AB, Stockholm, Sweden.

60. R. L. Olmon, B. Slovick, T. W. Johnson, D. Shelton, S.-H. Oh, G. D. Boreman, M. B. Raschke, Optical dielectric function of gold. *Phys. Rev. B* **86**, 235147 (2012).

61. N. W. Ashcroft, N. D. Mermin, *Solid state physics* (Cengage Learning, 2022).

62. D. Lengreth, *Many–Body Phenomena At Surfaces* (Elsevier, 2012).

63. A. Varas, P. García-González, J. Feist, F. J. García-Vidal, A. Rubio, Quantum plasmonics: From jellium models to ab initio calculations. *Nanophotonics* **5**, 409 (2016).

64. P. J. Feibelman, Microscopic calculation of electromagnetic fields in refraction at a jellium-vacuum interface. *Phys. Rev. B* **12**, 1319–1336 (1975).

65. R. C. Monreal, T. J. Antosiewicz, S. P. Apell, Diffuse surface scattering and quantum size effects in the surface plasmon resonances of low-carrier-density nanocrystals. *J. Phys. Chem. C* **120**, 5074–5082 (2016).

66. M. Svendsen, C. Wolff, A.-P. Jauho, N. A. Mortensen, C. Tserkezis, Role of diffusive surface scattering in nonlocal plasmonics. *J. Phys. Condens. Matter* **32**, 395702 (2020).

67. P. Pertsch, R. Kullock, V. Gabriel, L. Zurak, M. Emmerling, B. Hecht, Tunable nanoplasmonic photodetectors. *Nano Lett.* **22**, 6982–6987 (2022).
